# The effect of neighborhood social environment on prostate cancer development in black and white men at high risk for prostate cancer

**DOI:** 10.1371/journal.pone.0237332

**Published:** 2020-08-13

**Authors:** Shannon M. Lynch, Elizabeth Handorf, Kristen A. Sorice, Elizabeth Blackman, Lisa Bealin, Veda N. Giri, Elias Obeid, Camille Ragin, Mary Daly

**Affiliations:** 1 Cancer Prevention and Control, Fox Chase Cancer Center, Philadelphia, Pennsylvania, United States of America; 2 Department of Clinical Genetics, Fox Chase Cancer Center, Philadelphia, Pennsylvania, United States of America; 3 Cancer Risk Assessment and Clinical Cancer Genetics Program, Departments of Medical Oncology, Cancer Biology, and Urology, Sidney Kimmel Cancer Center, Thomas Jefferson University, Philadelphia, Pennsylvania, United States of America; San Diego State University, UNITED STATES

## Abstract

**Introduction:**

Neighborhood socioeconomic (nSES) factors have been implicated in prostate cancer (PCa) disparities. In line with the Precision Medicine Initiative that suggests clinical and socioenvironmental factors can impact PCa outcomes, we determined whether nSES variables are associated with time to PCa diagnosis and could inform PCa clinical risk assessment.

**Materials and methods:**

The study sample included 358 high risk men (PCa family history and/or Black race), aged 35–69 years, enrolled in an early detection program. Patient variables were linked to 78 nSES variables (employment, income, etc.) from previous literature via geocoding. Patient-level models, including baseline age, prostate specific antigen (PSA), digital rectal exam, as well as combined models (patient plus nSES variables) by race/PCa family history subgroups were built after variable reduction methods using Cox regression and LASSO machine-learning. Model fit of patient and combined models (AIC) were compared; p-values<0.05 were significant. Model-based high/low nSES exposure scores were calculated and the 5-year predicted probability of PCa was plotted against PSA by high/low neighborhood score to preliminarily assess clinical relevance.

**Results:**

In combined models, nSES variables were significantly associated with time to PCa diagnosis. Workers mode of transportation and low income were significant in White men with a PCa family history. Homeownership (%owner-occupied houses with >3 bedrooms) and unemployment were significant in Black men with and without a PCa family history, respectively. The 5-year predicted probability of PCa was higher in men with a high neighborhood score (weighted combination of significant nSES variables) compared to a low score (e.g., Baseline PSA level of 4ng/mL for men with PCa family history: White—26.7% vs 7.7%; Black—56.2% vs 29.7%).

**Discussion:**

Utilizing neighborhood data during patient risk assessment may be useful for high risk men affected by disparities. However, future studies with larger samples and validation/replication steps are needed.

## Introduction

In 2020, 191,930 new cases and 33,330 deaths from prostate cancer (PCa) are expected in the US [1]. Despite high survival rates, PCa remains the 2^nd^ leading cause of cancer death in US men [1]. Early detection of PCa through prostate specific antigen (PSA) testing is being re-visited to determine if PSA screening reduces PCa deaths [2] with mixed results [3]. PSA screening was not associated with reduced risk of prostate cancer mortality in both a US [4] and UK [5] randomized control trial, but was associated with reduced mortality in a European trial [6]. However, in the US trial, significant control group contamination (i.e., control group participants were still getting screened for PCa) [4], as well as low adherence to PSA screening in the UK trial [5], could have biased results towards the null [3]. Given the inconsistent findings and potential study biases, most professional organizations, including the American Urological Association [7], United States Preventive Services Task Force [8], American Cancer Society(ACS) [9], and National Comprehensive Cancer Network [10] generally recommend shared decision-making about undergoing PSA testing for men in the general population at average risk of PCa at age 55 up to age 69 (age 50 for ACS). However, the impact of PSA screening may be more substantial in high risk subgroups where the risk of dying from PCa is higher than expected [11]. Men with a PCa family history and Black men have a 2–7 fold increased risk for PCa compared to the general population [12, 13]. Further, Black men compared to White men are more than twice as likely to be diagnosed with and die of PCa [1], and are less likely to be screened for PCa [14]. These racial disparities in PCa outcomes have persisted for three decades [15, 16] and remain an important public health priority. However, the optimization of surveillance protocols and the effectiveness of current PCa screening guidelines for high risk men (i.e., shared decision-making about undergoing PSA testing at ages 40–54 in high risk men vs ages 55–69 in the general population [17]) continue to be understudied areas [18].

Identifying additional risk factors, beyond PSA, PCa family history, and race, could help to further risk stratify high risk men and improve clinical decision-making related to PCa screening and surveillance. Given the role racial disparities play in PCa outcomes, it seems plausible to also consider other measures of disparity, particularly socioeconomic (SES) determinants of health, such as a person’s education or income. As detailed by a number of multilevel conceptual frameworks [19, 20], beyond a person’s race/ethnicity or SES, social determinants of health, particularly macro-environmental factors, such as the social environment or neighborhood in which a person lives, may also impact cancer health disparities [20] and health more broadly [21]. Neighborhood or social environment is often defined in cancer studies by US Census variables related to socioeconomic status (SES) that describe the economic (e.g., employment, income), physical (e.g., housing/transportation structure), and social (e.g., poverty, education) characteristics of a census tract (smaller geographic boundary than a county) in which a person lives [22, 23]. Previous population-based studies of PCa found that neighborhoods defined by variables for low SES and higher deprivation were associated with greater risk of late-stage/high grade PCa in both Black and White men [22, 24, 25], and less aggressive treatment in Black men [22, 26]. Further, associations between neighborhood SES and PCa often remained, even after adjustment for race/ethnicity [22, 23]. Thus, given neighborhood is also correlated with access to care measures, such as screening utilization [27, 28], it is possible that social environmental variables could serve as novel metrics or environmental markers for high risk men on a disparity-related pathway to PCa.

The Precision Medicine Initiative calls for the consideration of multilevel risk markers—a person’s genes, lifestyle, AND environment—when making clinical decisions related to screening, treatment, and disease surveillance [29, 30]. Over 60% of PCa clinicians use risk assessment tools, such as nomograms to support clinical decision-making [31]. However, most PCa risk assessment tools focus only on patient-level clinical factors to identify which men are at risk for developing PCa or having poor outcomes [32–34]. More recently, an emphasis has been placed on the inclusion of genetic markers from genome-wide association studies (GWAS) [35–38], though validation of these GWAS markers is needed. Overall few, if any of these prediction studies considered social environment, and in some cases even patient-level racial/ethnic differences [31], despite well-known racial disparities in PCa.

Capitalizing on a novel neighborhood-wide association study (NWAS) we developed [39], we propose to comprehensively assess whether neighborhood social environmental (nSES) variables (from our NWAS study [39] and additional published literature [40–49]) are associated with time from study enrollment to prostate cancer diagnosis in an ethnically-diverse, longitudinal cohort of high risk men (e.g., Black men and those with a PCa family history) participating in an early detection prostate cancer risk assessment program (PRAP). In an exploratory analysis, we then assess whether social environment factors found to be significant in the proposed association study could inform the probability of developing PCa in high risk men. Thus, this study begins to bridge the gap between health disparity research at the population-level and the clinic by preliminarily assessing whether the incorporation of neighborhood data with patient-level clinical data could be useful for PCa clinical risk assessments.

## Materials and methods

### Study population

The Prostate Cancer Risk Assessment Program (PRAP) at Fox Chase Cancer Center (FCCC) in Philadelphia, Pennsylvania (PA) is a research, education, and screening program for men who are at high risk for PCa. It was established in 1996 to develop a registry of patients at high risk for PCa and to facilitate screening and early detection in these men [50]. As described previously [51], high risk men between the ages of 35–69 years and who were cancer-free at enrollment were recruited into the study [50]. High risk men were defined as: 1) having one or more first-degree relatives diagnosed with prostate cancer, 2) having 2 second-degree relatives on the same side of the family diagnosed with prostate cancer, 3) being of African American/Black race, or 4) having a known BRCA1/2 mutation [50]. Informed consent was obtained from all study participants for the PRAP cohort. This study was approved by the Fox Chase Cancer Center Institutional Review Board (Protocol #16–9007). Men were recruited into PRAP from the community through radio advertisements broadcast in the Philadelphia metropolitan area and from community partner hospitals [52]. PRAP participants are followed annually with clinic visits that include prostate specific antigen (PSA) testing and digital rectal exam (DRE) screening. Prior to November 2005, criteria for prostate biopsy included PSA > 4.0ng/mL or any abnormality on DRE. Given PCa detection rates were high in the cohort at this criteria, the criteria for prostate biopsy were lowered after November 2005 and included having a PSA ≥2.0ng/mL or any abnormality on DRE [51]. 73% of the PRAP cohort were recruited between 1996 and 2006. Within the PRAP cohort, 358 of 444 men residing in PA had complete residential address information, at least one follow-up visit, and were not missing relevant clinical (i.e., PSA, DRE) and demographic information (race/ethnicity; educational attainment) for analysis.

### Outcome

Our main outcome of interest was time to prostate cancer diagnosis. Men in PRAP were followed from time of study entry to PCa diagnosis or censoring (date of last study visit). The median follow-up time was over 55 months, with PCa occurring in 56 men.

### Patient-level factors

The following clinical factors were assessed for association with our outcome: age at baseline or enrollment (continuous variable), race/ethnicity (self-report White or Black), PCa family history (yes/no to having one or more first-degree relatives and/or 2 second degree relatives on the same side of the family diagnosed with PCa), PSA at baseline (ng/mL), and DRE at baseline (abnormal vs normal/benign prostatic hyperplasia [BPH]). Beyond these factors, we also evaluated models with and without a patient’s education level (high school or less vs. college education or more), given previously reported associations between education, PCa outcomes, health disparities, AND neighborhood circumstances [45–49]; however, education was not found to significant in any of our tested models.

### Social environmental factors

Neighborhood or social environment (nSES) is defined here by the economic (e.g., employment, income), physical (e.g., housing/transportation structure), and social (e.g., poverty, education) characteristics of a census tract in which a patient lives. Census tracts are typically smaller geographic boundaries than counties, containing on average 4000 residents. Neighborhood variables at the census tract level were ascertained from the Year 2000 US Census Summary File 1 (SF1) and Summary File 3 (SF3) data and downloaded via Social Explorer (http://www.socialexplorer.com) [53, 54]. Year 2000 census data were used because over 70% of the study participants were recruited between 1996 and 2006.

We previously designed a neighborhood-wide association study (NWAS), which is a multi-phase, empiric variable selection method derived from genome-wide association studies (GWAS) [39, 55] to agnostically analyze the independent association of over 14,500 US census variables and their association with aggressive PCa (Stage 3 or greater and Gleason grade ≥7) by race using the Pennsylvania State Cancer Registry data. We identified 22 variables (17 in White men; 5 in Black men) that were not highly correlated (S1 Table) and that remained significantly associated with aggressive PCa [39, 55]. In a subsequent replication study, 9 of 22 NWAS hits (4/5 from the NWAS in Black men; 5/17 from NWAS in White men) replicated across both Black and White men; whereas, 13 of the 22 variables appeared to be race-specific (S1 Table [56]) [57–59]. To further allow for a comprehensive investigation of social environment, we then coupled our NWAS findings [39] with findings from previous neighborhood and cancer studies [49, 57–59]. Thus, we identified and evaluated a total of 78 unique social environmental variables (e.g., nSES; representing the physical, social and economic landscape of Pennsylvania census tracts) for this analysis (see S1 Table).

Residential addresses of PRAP participants were geocoded up to the census tract level and assigned a Federal Information Processing Standard (FIPS) geocode [60, 61] at the census tract level using ArcGIS software v. 10.6. (ESRI; Redlands, CA). Patient information was then linked to the 78 variables representing nSES from the US census via the FIPS code using Stata v. 11.0 (College Station, TX). Thus, patients residing in the same census tract were assumed to have the same neighborhood characteristics. There were 235 unique census tracts included in this analysis.

### Statistical analysis

Distribution of study variables, including medians, ranges, and percentages were summarized overall and by race/ethnicity. nSES variables, age and PSA were evaluated as continuous variables. Variables with skewness >2 were log transformed; all variables were standardized to have a mean of zero and standard deviation of 1.

#### Association study

We assessed the relationship between all 78 nSES variables and time to PCa diagnosis using a series of univariable Cox proportional hazards regression models. Census variables with P<0.10 were included in a subsequent multivariable Cox model along with patient-level factors (age, DRE, PSA, race, and family history), using robust standard errors to account for clustering within census tract (S1 Table). Hazards ratios, 95% Confidence Intervals, and p-values (significance at p-value <0.05) from refined multivariate models (that only include significant neighborhood variables to reduce potential over-fitting) are reported. Due to the ascertainment strategy of the PRAP cohort and to account for confounding, we also divided the sample by race, and assessed the nSES variables which were applicable to the given racial group (S1 Table). The Black population was further subdivided by family history. We evaluated model fit/performance of patient-only factors versus combined models by comparing AIC estimates [62].

As a secondary analysis, we also used the lasso machine learning method [63, 64] to assess whether nSES variables were predictive of time to PCa diagnosis. This analysis was used 1) as an alternative variable reduction/selection technique, as it can accommodate correlation amongst nSES factors, given census variables are often highly correlated with one another and race/ethnicity [39]; 2) to confirm results from our association study, i.e., to demonstrate whether the same neighborhood variable could be identified across multiple methods, which would be relevant for the preliminary assessment of clinical relevance (described below). Variables with non-zero coefficients were considered to be of interest, with the penalty parameter chosen via cross-validation.

#### Preliminary assessments of clinical utility

Next, we calculated the predicted 5-year rates of PCa diagnosis for each participant based on the coefficients of the Cox regression models. Similar to genetic risk score calculations, we then calculated a neighborhood exposure score as the weighted sum of the neighborhood covariates (values weighted by the regression coefficients) in the total population and each race-specific group [65]. Neighborhood exposures were dichotomized at the median to categorize participants as residing in a neighborhood with either high or low exposure to identified nSES conditions. For each individual, the 5-year predicted probability of being diagnosed with PCa was then plotted against baseline PSA level, grouped by high/low neighborhood exposure score, to preliminarily demonstrate potential clinical utility.

## Results

Baseline characteristics of the PRAP cohort are presented in Table 1. The median age at study entry was 50 years old; 56% of the study cohort included Black men. The median baseline PSA levels were 0.95ng/mL, and the percentage of patients with a normal DRE was 96.9% for the total sample. The median duration of follow-up was close to 5 years, with Black men having longer median follow-up than White men (60 vs 50.7 months, respectively). 42% of Black men report having a PCa family history. Black men were also more likely to be diagnosed with PCa (18.9% vs 10.8%) and to have a high school education or less (35.3% vs 19.5%) compared to White men. Black men also lived in neighborhoods with a higher percentage of persons living below poverty (median = 16%) compared to White men (median = 3.7%). Approximately 60% of Black men and 36% of White men lived in a neighborhood with high exposure to nSES circumstances associated with shorter time to prostate cancer in this analysis. Men in the high neighborhood exposure group (i.e., those exposed to significant social environmental conditions) compared to those in the low exposure group had a higher percentage of men diagnosed with PCa (21% vs 10%) and advanced PCa (Gleason grade ≥7) (29% vs 13%).

**Table 1:** Baseline characteristics of 358 men by self-report race.

	Total Population	Black	White
	(n = 358)	(n = 201)	(n = 157)
Age at entry—median years (range)	50 (35–69)	51 (35–68)	49 (35–69)
Duration of follow-up- median months (range)	55.1 (0.1–222.9)	60.0 (0.1–213.0)	50.7 (0.1–222.9)
Have a high school education or less	28.5% (n = 102)	35.3% (n = 71)	19.7% (n = 31)
% Prostate Cancer Family History (N)	67.3% (n = 240)	42.3% (n = 85)	100% (n = 157)
Median PSA (ng/mL) at baseline (range)	0.95 (0.1–9.8)	1.0 (0.1–7.9)	0.90 (0.2–9.8)
DRE* at Baseline			
% Normal/BPH** (N)	96.9% (n = 347)	96.5% (n = 194)	97.5% (n = 153)
% Abnormal (N)	3.1% (n = 11)	3.5% (n = 7)	2.5% (n = 4)
% Prostate Cancer Diagnosis (N)	15.4% (n = 56)	18.9% (n = 38)	10.8% (n = 17)
% of Patients Living in Neighborhood with High Exposure to Significant Neighborhood Socioeconomic variables (N)***	49.7% (n = 178)	59.7% (n = 120)	36.9% (n = 58)

*DRE = Digital Rectal Exam

**BPH = Benign Prostatic Hyperplasia

***High Neighborhood Exposure Score was calculated as the weighted sum of the final significant neighborhood socioeconomic variables (nSES; values weighted by the penalized coefficients from the final patient plus neighborhood-level models) in the total population and each race-specific group. Neighborhood exposures were dichotomized at the median to categorize participants as residing in a neighborhood with either high or low exposure to significant neighborhood socioeconomic conditions.

### Association study

In the full study sample and in race/PCa family history-specific models, nSES variables were associated with time to PCa diagnosis, along with patient and clinical characteristics, but the findings did differ across race/ethnic groups (Table 2 and S2 Table). For this reason and due to the ascertainment strategy (i.e., recruiting Black men and White men with a PCa family history) we focus on results in stratified models. In multivariate models, as expected PSA was strongly associated with time to PCa diagnosis across all statistical models (HR = 1.47; 95%CI = 1.33–1.63 p-value = <0.001) (Table 2). A neighborhood housing variable (% Owner-occupied housing units with 3 or more bedrooms) was consistently associated with a shorter time to PCa diagnosis in the total study population and in Black men with a PCa family history. In Black men without a PCa family history, % unemployed workers was associated with time to PCa diagnosis (HR = 1.80; 95%CI = 1.11–2.92 p-value = 0.02). In White men, an employment/ transportation variable (% Workers taking trolley or street cars to work), which was the “top” hit in the previous NWAS study, was also associated with time to PCa diagnosis (HR = 2.50, 95%CI = 1.33–2.866; p-value <0.01). A low income variable (% Males with annual income of $7500–9999; HR = 1.81, 95%CI = 1.27–3.44, p-value = 0.004) was also significant in White men.

**Table 2:** Significant patient and neighborhood variables in final multivariate cox models in the total study population and by race/ethnicity/PCa* family history.

	Total Population n = 358	White (w/ PCa Family Hx*) n = 157	Black (w/ PCa Family Hx) n = 85	Black (without PCa Family Hx) n = 116
	Multivariable Model (HR 95%CI p-value±)	Multivariable Model (HR 95%CI p-value±)	Multivariable Model (HR 95%CI p-value±)	Multivarible Model (HR 95%CI p-value±)
**Significant Neighborhood Factors (variable name)**				
% Workers Taking Trolley or Street car transportation to work (PCT_SF3_p030007)		2.50	.	.
		1.33–2.85;		
		<0.01		
% Black Males in the Population (PCT_SF1_P012B002)	0.65			
	0.48–0.94			
	0.02			
% Owner-occupied housing units with 3 or more bedrooms (PCT_SF3_H042006)	1.46		1.76	.
	1.11–1.94		1.12–2.77	
	0.008		0.01	
% Males with earnings of $7,500–9,999 (PCT_SF3_p084006)	.	1.81	.	.
		1.27–3.44		
		0.004		
% Unemployed in the Labor Force (PCT_SE_T069_y)	.	.	.	1.80
				1.11–2.92
				0.02
**Patient-Level Factors**				
Baseline Age	1.03	1.05	0.99	1.05
	1.00–1.07	0.99–1.12	0.93–1.05	0.97–1.15
	0.07	0.07	0.67	0.23
Black Race	2.02	.	.	.
	1.01–4.23			
	0.04			
Prostate Specific Antigen (PSA) ng/mL	1.47	1.37	1.81	1.90
	1.33–1.63	1.18–1.60	1.50–2.19	1.23–2.93
	<0.001	<0.001	<0.001	0.003
Digital Rectal Exam	1.04	3.70	0.71	.
	0.27–3.68	0.58–5.69	0.12–4.34	
	0.94	0.17	0.71	
PCa Family Hx	1.49	.	.	.
	0.79–2.88			
	0.23			

* PCa Family Hx = Prostate Cancer Family History

^±^Hazard Ratio (HR), 95% Confidence Intervals presented are based on the hazard of an event where HRs greater than 1 reflect clinically worse outcomes (i.e., associated with shorter time to diagnosis), and HRs less than 1 represent clinically better outcomes (i.e., associated with longer time to diagnosis);

“^.^” signals this variable was not assessed due to lack of significance in univariable or multivariable models

Lasso results were similar to variables identified with univariate analysis (S2 Table). For patient level variables, race, PSA at baseline, and age had non-zero lasso coefficients, but family history and DRE did not. We also explored interactions with race, but no interactions were non-zero. Neighborhood variables found to be significant in multivariable models also had non-zero penalized coefficients in lasso (e.g., pct_sf3_p030007 [coefficient = 1.01] and pct_sf3_p084006 [coefficient = 0.72] for the White population; pct_sf3_h042006 [coefficient = 0.14] for Black participants with PCa family history; and pct_SE_T069_y [coefficient = 0.14] for Black men without a PCa family history). In both lasso and cox regression models, model fit was improved in combined models (patient plus neighborhood variables) compared to models with only patient level data (S2 Table). In cox regression, the AIC for patient models compared to combined models for the total population was 528.18 vs 519.52; for White men with a PCa family history 143.38 vs 131.73; for Black men with a PCa family history 149.44 vs 144.95; and for Black men without a PCa family history 110.87 vs 107.51. Moving forward, we chose to work with the Cox regression findings from the combined models (Table 2), given the significant neighborhood variables identified in these models were found to be informative across both regression and lasso methods.

### Preliminary assessments of clinical utility

**Fig 1:**
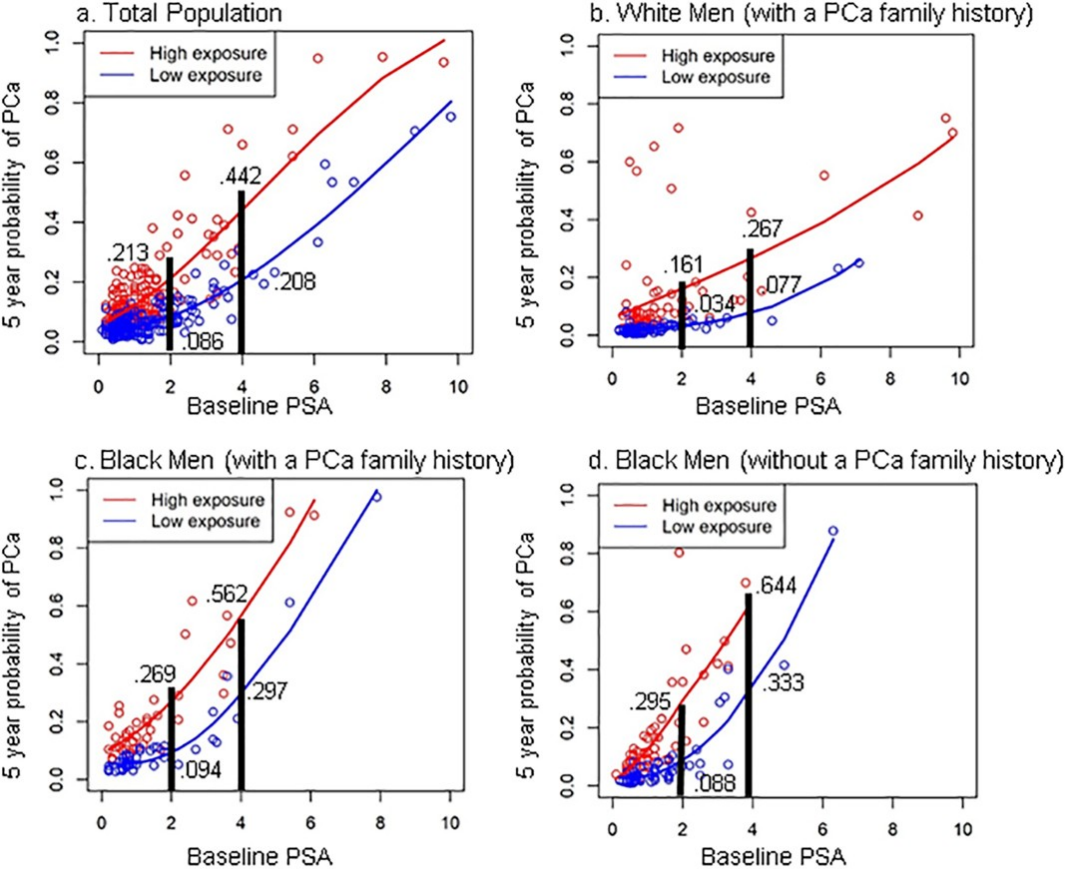
A-D. Five-Year Predicted Probability of Prostate Cancer (PCa) by Baseline PSA Level and High/Low Neighborhood Exposure Score. (A) Total population. (B) White men (with a PCa family history). (C) Black men (with a PCa family history). (D) Black men (without a PCa family history).

Utilizing the findings from combined (patient plus neighborhood) Cox models above, we calculated the 5-year predicted probability of a PCa diagnosis, and it was higher in men from social environments with a high neighborhood exposure score (high exposure to weighted combinations of significant nSES variables from the multivariable model) compared to those with a low exposure score (low exposure to weighted combinations of significant nSES variables from the multivariable model) in the total population and in White and Black men with/without a PCa family history (Fig 1A–1D). Differences in predicted probabilities were most notable for participants with larger PSA values at baseline. For example, White men with a PCa family history residing in the high neighborhood exposure group with a baseline PSA level of 4ng/mL had a 26.7% (compared to 7.7% from the low exposure group) 5-year predicted probability of PCa. For Black men with a family history residing in the neighborhood high exposure group, the 5-year predicted probability of PCa was 56.2% versus 29.7% in the low exposure group (Fig 1C). For Black men without a family history of PCa, the 5-year predicted probability of PCa was 64.4% (extrapolated) in the high neighborhood exposure group, and 33.3% in the low neighborhood exposure group (Fig 1D).

## Discussion

This study is one of the first to consider both the association between neighborhood or social environmental determinants and time to PCa diagnosis in a clinical sample of high risk men, as well as to assess the potential clinical utility of nSES variables. Time to PCa diagnosis is a relevant outcome to study, given the increased interest in evaluating the effectiveness of current PCa screening guidelines for high risk men (i.e., Black men or those with a PCa family history), and the growing interest in improving surveillance protocols for men who choose “watchful waiting”[17, 18]. Neighborhood SES factors are important to consider because they are implicated in PCa health disparities, specifically differential diagnosis of PCa risk and survival outcomes and differential access to care [40–49]. Given neighborhood social environment could serve as an additional indicator of poor PCa outcomes, it is possible they could also impact clinical outcomes such as time to PCa diagnosis. Thus, investigations into whether neighborhood can improve the identification of men at high risk for PCa, particularly those who may be impacted by disparities, seem plausible. However, neighborhood findings are rarely tested in clinical settings, where more detailed patient and clinical data (that might correlate with or fully account for neighborhood effects) are available. In a clinical cohort of men at high risk for PCa (PRAP), our initial study suggests that when jointly evaluated social environmental factors remain associated with time to PCa diagnosis, even in the context of patient clinical factors. However, findings were slightly different by race/ethnicity. Further, the incorporation of neighborhood data appeared to modestly improve the five-year predicted probability of being diagnosed with PCa in high risk men (Black race and/or PCa family history), suggesting continued investigations into the clinical utility of social environmental factors are needed.

### Association study

We took a comprehensive approach and analyzed many nSES variables because across cancer studies, different neighborhood variables are often selected to represent the same general SES domains (e.g., education, income, employment). For instance, one study might define poverty in terms of income, another in terms of percentage of households above or below the State poverty line. This has complicated conclusions about the relationship between neighborhood and cancer outcomes [59] and has made the translation of neighborhood findings to the clinic challenging. Therefore, in this association study, we used patient clinical variables that have been shown to affect time to PCa diagnosis [36, 51], and neighborhood variables that were previously found to be associated with health outcomes across the cancer continuum (incidence, aggressiveness, mortality) (S1 Table). Thus, the patient and neighborhood-level variables identified in multivariate models are also biologically plausible in that they have previously been shown to be associated with poor outcomes. However, census variables across SES domains are often correlated [39], and our comprehensive approach to include multiple variables also introduces possible multicollinearity. To address these challenges, we applied empiric methods approaches to allow for systematic variable selection (univariate analysis and prior NWAS) and to account for correlation within the data prior to building the multivariable models (using lasso). Given the study ascertainment strategy and potential for confounding by race[1, 66], we focus on interpretation of results in stratified models.

Prior PCa studies have found associations with nSES extremes (measured in terms of education, employment, and poverty variables [58, 67]). More specifically, both high and low nSES have been reported to have associations with poor PCa outcomes [22, 39, 56, 67, 68]. Low SES is often associated with increased PCa stage or grade and increased mortality [22, 39, 56, 69, 70]; whereas studies of PCa incidence have found associations with higher SES, partially attributed to increases in PCa screening in men from higher socioeconomic groups [67, 68]. Similarly, in this study, we also found significant associations between time to PCa diagnosis and low nSES variables. Although numbers were small, men with high exposure to these low nSES variables also accounted for the majority of advanced PCa diagnoses (12 of the 14 Gleason grade ≥7 diagnoses). Specifically, we found that in Black men without a PCa family history, neighborhoods with a higher percentage of unemployment were associated with shorter time to PCa diagnosis [69, 70]. In White men with a PCa family history, neighborhoods with a higher percentage of men with low incomes were also associated with shorter time to PCa diagnosis [22]. A joint domain variable related to employment and transportation was also identified in White men (%workers taking trolley or street cars to work). This variable (pct_sf3_p030007) was positively correlated with poverty-like variables (S1 Fig/S3 Table), relates to urban compared to rural environments, and can be used as a surrogate measure for access to care [71, 72].

In Black men with a PCa family history, one nSES variable related to housing and homeownership (i.e., owning a home with greater than 3 bedrooms) was associated with shorter time to diagnosis of PCa. In prior literature, housing factors, particularly renting a home and overcrowding (i.e., having more than one occupant per room), both of which often correlate with lower SES and unequal housing policies, have been shown to contribute to disparities in the Black community [56, 68, 73, 74]. Thus, our finding that housing related to homeownership was associated with a poor PCa outcome was surprising. To further understand this variable, we reviewed correlations with other variables in our study population (S1 Fig). We found the highest correlations with this variable (range 0.41–0.58) were with variables representing favorable housing conditions (i.e., owner-occupied, single family units) and blue collar occupations (i.e., % working in construction and/or male public service occupations); however, inconsistent correlations were observed with income and poverty variables (e.g., negative correlations reported with both high income >$150K and low income <$15K) (S1 Fig). Thus, the homeownership variable, pct_sf3_h042006, may reflect living in a middle class neighborhood.

Few studies in cancer have specifically evaluated the effect of middle class socioeconomic status on cancer outcomes, particularly in race-specific analyses. PCa incidence studies have found associations with higher neighborhood SES (measured as a composite SES score that included variables related to education, employment, and income) [67, 68]. The positive associations reported for nSES and PCa incidence in these studies have been attributed to access to care, suggesting men with higher SES are more likely to get PCa screening and get diagnosed at lower stage disease [67, 68]. However, in our study cohort, participants were enrolled in an early detection program, where all participants received annual PSA screening and subsequent biopsies, with some receiving more aggressive screening (e.g. at younger ages) and biopsies (e.g. based on PSA of 2ng/mL) than recommendations specify for a general population. When screening is broadly accessible, associations between race/ethnicity [75], high nSES and PCa outcomes have been shown to attenuate [67]. Thus, it’s possible our low and mid-level SES findings were impacted by the availability of screening. Additionally, in one of the prior PCa incidence studies using California (CA) cancer registry data, the middle quantiles (Q) of nSES (i.e., Q3 and Q4 from Q1-Q5), which likely represent middle class status, were also significant in Black and White men [68]. However, in this study, important clinical factors, including PSA and PCa cancer family history were not available. In another PCa incidence study in Detroit, a matched case-control design was employed, and the analysis included adjustments for PCa cancer family history [67]; however, nSES was assessed continuously and cases and controls were matched by race, so associations with mid-level SES alone and by race could not be ascertained. Further, this study also used a ZIP-code level nSES composite score [67] compared to smaller census tract level nSES measures used in our study and the study conducted in CA. Level of geography has been shown to affect study results [76]. Additionally, a limitation of prior studies is the use of a nSES composite score that might include variables that are not relevant for specific race/ethnic groups [68]. This is one of the reasons we evaluated each nSES variable separately [56]. Our study population contained more Black men and men with a PCa family history, tended to be younger, and varied geographically (our patients were from PA compared to CA and Detroit) [67, 68], which could also influence findings. Further, previous studies show that certain racial/ethnic groups at the same level of SES may not share the same level of power or opportunities, and it’s possible that the race-specific nSES variables identified in this study may provide insight into this phenomenon [77]. Thus, race-specific findings warrant further study [73], as most cancer studies focus on general neighborhood effects with less emphasis on which/why social environmental factors may be different across race/ethnic groups [40–49]. Further, it is also possible that neighborhood factors not included in this analysis, particularly variables related to access to care, such as distance/travel time to health care facilities [78], could also impact time to PCa diagnosis [79, 80]. In general, this study provides additional evidence that nSES can impact time to PCa diagnosis; however, these relationships are complex and additional studies are needed to further explore the direction of the association, in the context of race/ethnicity.

Our study also showed interesting differences within Black men based on family history (i.e., associations with shorter time to PCa diagnosis were found with a moderate nSES variable in Black men *with* a PCa family history AND with low nSES (i.e., unemployment) in Black men *without* a family history). Family history of PCa is strongly influenced by genetics [79, 80], and it is possible that genetics or tumor biology play a strong role in poor PCa outcomes [73, 74]. Similarly, neighborhood environment compounded with underlying genetic susceptibility may also affect PCa development. Studies do show differences in molecular mechanisms for PCa by race [81]. Further, neighborhood circumstances have been found to affect DNA repair pathways that are also implicated in PCa family history [82–84]. However, under a chronic stress hypothesis, it is also possible that residents from disadvantaged neighborhoods may experience greater emotional stress and constant “wear and tear” on the body that can influence not only DNA repair, but epigenetic changes that can ultimately result in cancer development [85–89]. Thus, although our interpretations are limited because we have a small sample of Black men without a PCa family history, and we lack a comparison group of White men without a PCa family history, our findings do suggest that future cancer investigations centered on gene and social environment interactions and social epigenetic changes by race appear warranted.

While a handful of neighborhood variables were identified in the association study, 3 of 4 significant nSES variables from race-stratified models came from our previous empiric NWAS studies [39, 56]. NWAS variables tend to be more specific (i.e., gender/age/specific income range) and represent more than one domain in a single variable compared to traditional, single domain variables (i.e., % unemployment). While it is possible that findings could be due to chance, this is unlikely given that across multiple methods (S2 Table) and study populations (the PRAP population here and the PA cancer registry from the previous NWAS [56]), % of owner-occupied housing units with 3 or more bedrooms in Black men, and % of workers taking trolley or street cars to work and % of males earning low income in White men) continue to replicate and show associations with PCa outcomes. Thus, coupling NWAS approaches with commonly used single domain variables may be informative in future studies.

### Preliminary assessments of clinical utility

Multivariate models that contained both patient and neighborhood variables modestly improved model fit for time to PCa diagnosis compared to patient only models across race/ethnic groups. Similarly, the 5-year predictive probability of developing PCa was higher in men from neighborhoods with a higher nSES exposure score compared to men from neighborhoods with a lower neighborhood exposure score. The neighborhood exposure scores were derived from significant nSES variables from the association models, and this effect (i.e. higher exposure scores or higher percentages of significant nSES variables) remained regardless of the direction of the nSES variable (i.e. low SES or mid-level SES), and was more pronounced in men with higher PSA values. If this finding is validated in large independent datasets, it could potentially help identify participants from geographic areas at particularly high risk of developing PCa. For instance, the 5-year predicted probability of PCa was close to triple for White men (PCa family history) with a baseline PSA of 4ng/mL who lived in neighborhoods with high exposure to unfavorable socioeconomic circumstances (26.7%) compared to men with the same PSA level from low exposure neighborhoods (7.7%). The probability was 5 times greater for White men with a PCa family history and a baseline PSA of 2ng/mL living in a high exposure neighborhood (16.1%) compared to a low exposure neighborhood (3.4%). Similarly, Black men with a PCa family history from high exposure neighborhoods who had a baseline PSA of 4ng/mL had close to double (closer to triple at a PSA of 2ng/mL; 26.9.2% vs 9.4%) five-year predicted probability of PCa. In Black men without a PCa family history, we had to extrapolate the high neighborhood score at a PSA of 4ng/mL due to sample size (i.e., few men with a PSA of 4ng/mL lived in areas with higher percentages of unemployment). However, patterns were similar to the other stratified analyses at a PSA of 2ng/mL. Black men without a PCa family history who had higher neighborhood exposure scores had greater than 3 times the five-year predicted probability of PCa (29.5% vs 8.8%). We chose to evaluate the 5-year probability of developing PCa at the 4ng/mL and 2ng/mL PSA cutpoint, since this is the value often used to determine whether to move forward to a prostate biopsy or continue active surveillance in PRAP participants [51]. Prostate cancer has a long latency period and we would have liked to assess the probability of developing PCa at longer time intervals (i.e., 10 or 15 years); however we chose to evaluate the probability of developing PCa at 5 years post enrollment, given the majority of our study sample had follow-up of at least 5 years. Thus, while findings preliminarily suggest the incorporation of social environmental factors might be useful for screening and active surveillance decisions, more studies are needed, given we could not evaluate the probability of developing PCa over a longer time period due to loss of follow-up, and the majority of the study sample did have a PCa family history.

## Conclusions

There has been a recent increase in both the recognition and importance of utilizing social determinants of health to improve cancer treatments and interventions [21]. In line with the Precision Medicine Initiative’s emphasis on environment [29, 30], to our knowledge, this study is one of the few to investigate the association of neighborhood or social environment with time to PCa diagnosis in a clinical sample of high-risk men. While this study introduces new systematic and empiric methods that can inform future PCa neighborhood and clinical studies, it was not an etiologic study designed to conclusively identify the most important neighborhood factors impacting PCa risk. Further, the cross-sectional assessment of neighborhood circumstances only at the time of enrollment, the modest sample size (particularly when stratifying by race and PCa family history), the study ascertainment strategy, the lack of availability of additional patient level clinical data (i.e., repeated measures of PSA) as well as patient SES data [90], and the limited generalizability of our findings due to the high proportion of men with PCa family history, limit our conclusions. However, our results can serve to be hypothesis-generating for future investigations into the role of PCa health disparities in the clinic. Finally, preliminary findings from this limited sample demonstrate a proof of concept that predicted probability of PCa may be augmented with the addition of publically-available, readily accessible nSES factors from the US Census. While additional studies with larger sample sizes and additional validation and replication steps are needed, findings suggest that neighborhood variables could potentially add useful clinical information for high risk men undergoing PCa risk assessment.

## Supporting information

S1 Table List of neighborhood variables for analysis. Includes NWAS census variables that represent neighborhood disparities (socioeconomic status (SES), social support, physical environment/access) associated with initial diagnosis of aggressive prostate cancer (stage ≥3/Gleason ≥7).
(DOCX)

S2 Table Univariate analysis and LASSO replication findings model fit criteria (AIC, BIC). (DOCX)

S3 Table Correlation analysis of neighborhood socioeconomic variables. (DOCX)

S1 Fig Correlation analysis of neighborhood socioeconomic variables. (DOCX)
