# Supplementary material for: The effect of neighborhood social environment on prostate cancer development in black and white men at high risk for prostate cancer
Source: PLoS One. 2020 Aug 13;15(8):e0237332. doi: 10.1371/journal.pone.0237332 (PMC7425919; doi:10.1371/journal.pone.0237332)
Supplement: S1 Table — Includes NWAS census variables that represent neighborhood disparities (socioeconomic status (SES), social support, physical environment/access) associated with initial diagnosis of aggressive prostate cancer (stage ≥3/Gleason ≥7). (DOCX) [file pone.0237332.s001.docx]

**Table S1. List of Neighborhood Variables for Analysis**

1. **Neighborhood-wide Association Study variables (NWAS) Census Variables that Represent Neighborhood Disparities (Socioeconomic Status (SES), Social Support, Physical Environment/Access) Associated with Initial Diagnosis of Aggressive Prostate Cancer (Stage >3/Gleason >7)** (references 33, 39, and 56)

**Name Description Disparity-Related Domain**

1. pct_sf3_pct075a006 %White alone population for whom poverty status is determined age 6-11 years SES/poverty/Race/Age
2. pct_SF3_p159i007 %White, Non-hispanics where poverty status determined aged 18-64 below SES/poverty/Race/Age

poverty level in 1999

1. pct_sf3_p092021 % Male Nonfamily households below poverty level SES /poverty/gender
2. pct_SF3_h019093 %Male householder living alone (nonfamily household) Social Support/gender
3. pct_SF3_hct005083 % Renter occupied housing unit built 1939 or earlier with householder aged 15

24 years Physical Environment/age

1. pct_sf3_p120002 Imputed civilian non-institutionalized population 5 years and older
2. pct_SF3_p052012 %Household income $60K-74,999 SES/income
3. pct_SF3_pct051020 %Foreign born naturalized citizen at or above poverty level SES/immigration
4. pct_SF3_hct017019 %Household income of $10K-19,999 with owner-occupied housing unit value of SES/housing/income

$10K-19,999

1. pct_sf3_pct050102 % Population for whom poverty status is determined aged 45-54 years, under SES/age/poverty

0.50

1. pct_sf3_hct015042 % Aggregate income of Occupied Housing units built 1940-1949 SES/housing
2. pct_SF3_p030007 %Workers 16 years and over taking public transportation, namely trolley or Employment/Access/Middle Class SES

street cars, to work

1. pct_SF3_h045025 Renter occupied housing unit with householder aged 55-64 with no vehicle Access/housing/age

available

1. pct_SF3_p050026 %Male Protective Service Occupations: fire fighting, prevention, and law SES/employment/gender

enforcement workers

1. pct_SF3_p084006 %Males with earnings of $7500-9,999 in 1999 SES/income/gender
2. pct_SF1_p030012 %Male householder over 65 living alone in nonfamily household Social Support/gender
3. pct_sf3_hct004093 % Household Renters aged 55-64 years SES/housing/age
4. pct_SF3_H021045 % Renter-occupied housing unit with householder age 45-54, with 1.01-1.5 SES/Housing(crowding)/age/

occupants per room (crowding)

1. pct_SF3_H111002 Imputation of kitchen facilities SES/Housing
2. pct_SF3_H042006 Owner-occupied housing unit with 3 bedrooms Housing/employment/middle class
3. pct_SF3_PCT025051 Females 18-24 with a graduate or professional degree SES/education/gender
4. pct_SF3_P033003 Workers 16 and over who do not work at home, aggregate travel time to work Employment/Access/Urban

less than 30 minutes by public transportation

Highlight indicates this variable replicated in both Black and White men.

1. **Standard Census Variables** from literature (references 50-51)

**Name Description Disparity-Related Domain**

1. PCT_SF3_P010015 % Female-headed households with kids SES/social support
2. PCT_SE_T059_002 % High school dropouts 16-19 years old SES/education
3. Popzeronine % Population aged 0-9 years Younger Ages
4. Poptennineteen % Population aged 10-19 years Younger ages
5. SF3_P053001 Median household income SES/income
6. SF3_H085001 Median value owner-occupied home SES/housing
7. Novehicle % No vehicle available Access/transportation
8. PCT_SF3_P087002 % Persons below poverty line SES/poverty
9. PCT_SF1_P007003 % Population black Race
10. PCT_SF1_P007002 % Population white Race
11. PCT_SF1_P010009 % Population Hispanic Race
12. PCT_SE_T069_006_Y % Unemployed, labor force aged 16+ years SES/employment
13. Collegeover25 % With college degree, age 25+ years SES/education
14. PCT_SE_T040_002 % With no high school diploma, age 25+ years SES/education
15. PCT_SE_T085_017 % Managerial/professional/technical occupation SES/employment
16. PCT_SF3_P018013 % Females aged 15+ married Social Support
17. PCT_SF3_P021013 % Persons foreign born SES/immigration
18. PCT_SF1_P037002 % Persons institutionalized SES
19. Houses1970 % Housing built since 1970 Housing/Physical Environment
20. PCT_SF3_H034009 % Housing units built pre-1940 Housing/Physical Environment
21. PCT_SF3_H038002 % Housing units owner occupied SES/housing
22. PCT_SF3_H047003 % Housing units lacking plumbing SES/housing
23. PCT_SF3_P115007 % Aged 5+ years in same unit 5+ years SES (mobility)
24. PCT_SE_T159_002 % Units in single-family structure Housing/Physical Environment
25. Vacanthouse % Housing units vacant OR SF1_H003003 SES/Housing
26. PCT_SE_T027_002 % Population Never Married Social Support
27. PCT_SE_T027_003 % Population Married Social Support
28. Hscollegegrad % adults with high school or college graduate SES/education
29. Morecollege % adults with greater than a college degree SES/education
30. PCT_SE_T069_005 %16 and over in civilian labor force, employed SES/employment
31. PCT_SE_T070_002 %16 Male population 16 and over in labor force SES/employment
32. PCT_SE_T070_006 %16 and over in civilian labor force, unemployed SES/employment
33. Workingclass %Working class occupation SES/employment
34. PCT_SE_T086_002 % Management, business, and financial operations employment SES/employment
35. PCT_SE_T086_003 % Professional and related occupation SES/employment
36. Bluecollar % Blue Collar Occupation SES/ employment
37. PCT_SE_T086_012 % Construction, extraction, and maintenance occupations SES/employment
38. PCT_SF1_P020012 %Female head of householder with no husband and children under 18 below SES/poverty

poverty

1. PCT_SF1_P003004 %Population Black alone Race/ethnicity
2. PCT_SF1_P010010 % White Hispanic or Latino Race/ethnicity
3. PCT_SF1_P010011 % Black Hispanic Race/ethnicity
4. PCT_SF1_P012B002 % Black Males Race/Gender
5. PCT_SF1_H003003 %Vacant Housing Units Housing/Physical Environment
6. PCT_SF3_P064002 % Households with public assistance income SES/income
7. HHincome15K % Household income less than $15K SES/income
8. HHincome150K % Household income greater than 150K SES/income
9. PCT_SF3_P052002 % Households less than $10K SES/income
10. PCT_SF3_P052003 % Households $10K to 15K SES/income
11. SF3_P077001 Median family income SES/income
12. PCT_SF3_P089002 % Individuals with income below poverty SES/poverty
13. PCT_SF3_P089021 % Individuals with income at or above poverty level SES/poverty
14. PCT_SF3_P090002 % Families with income below poverty SES/ poverty
15. PCT_SF3_P092002 % Households with families with income below poverty SES/poverty
16. PCT_SF3_H020002 %Occupied housing units that are owner occupied SES/housing
17. Crowding %homes with >1 person per room SES/housing
18. Homeprice % Owner-occupied homes greater than $300K SES/housing

**Note: Nomenclature**. The neighborhood or social environmental variables are derived from the US census.  In general, these variables start with the prefix:

SF1_

SF3_

PCT_SF1_

PCT_SF3_

*Note, after the prefix, the following number corresponds to the Census codebook.  For instance, in the SF1 codebook, variable P002002 (% of total population living in urban environment) would correspond to PCT_SF1_P002002 in our dataset.  Here is the SF1 codebook for this data:  <https://www.census.gov/prod/cen2000/doc/sf1.pdf>. Here is the SF3 codebook:  <https://www.census.gov/prod/cen2000/doc/sf3.pdf> . Variables with “_SE” in the name or without a pct_ prefix are calculated variables using combinations of existing census variables.
