# Supplementary material for: The effect of neighborhood social environment on prostate cancer development in black and white men at high risk for prostate cancer
Source: PLoS One. 2020 Aug 13;15(8):e0237332. doi: 10.1371/journal.pone.0237332 (PMC7425919; doi:10.1371/journal.pone.0237332)
Supplement: S2 Table — (DOCX) [file pone.0237332.s002.docx]

**S2 Table. Univariate Analysis and LASSO Replication Findings Model Fit Criteria (AIC, BIC)**

1. **Univariate Analysis and LASSO Results**

Univariate: Cox Regression Models, reporting Hazards Ratios (HR), 95% confidence intervals (CI) and p-values.

Lasso: We used the R package glmnet to fit the Lasso models,^1^ and we evaluated model performance using: 1) the Akaike Information Criterion (AIC) ; 2) predictive ability evaluated by the area under the receiver operating curve (AUC).^2^

1. Friedman J, Hastie, J., Tibshirani, R. Regularization Paths for Generalized Linear Models via Coordinate Descent. Journal of Statistical Software. 2010;33(1):1-22.
2. Heagerty PJ, Lumley, T., Pepe, M.S. Time-dependent ROC Curves for Censored Survival Data and a Diagnostic Marker. Biometrics. 2000;56:337-44.
3. **Total Study Population**

|  | coef | HR | se(coef) | z | Pval | 95% CI |  | *P-value <0.10; tried in multivariate model (also found in Lasso) |
| --- | --- | --- | --- | --- | --- | --- | --- | --- |
| pct_sf3_p030007 | 0.217317 | 1.242738 | 0.102285 | 2.124629 | 0.033618 | 1.016982 | 1.51861 | * (LASSO) |
| pct_sf3_p050026 | 0.122183 | 1.129961 | 0.131574 | 0.928626 | 0.353083 | 0.873103 | 1.462385 |  |
| pct_sf3_p052012 | 0.005447 | 1.005462 | 0.140045 | 0.038894 | 0.968975 | 0.764111 | 1.323045 |  |
| pct_sf3_p084006 | 0.20727 | 1.230315 | 0.135498 | 1.529692 | 0.126093 | 0.943361 | 1.604554 |  |
| pct_sf3_p092021 | 0.03083 | 1.03131 | 0.127177 | 0.242419 | 0.808456 | 0.803774 | 1.323258 |  |
| pct_sf3_p120002 | 0.158008 | 1.171176 | 0.134963 | 1.170755 | 0.241697 | 0.898958 | 1.525825 |  |
| pct_sf3_p159i007 | 0.142453 | 1.153099 | 0.124399 | 1.14513 | 0.252155 | 0.9036 | 1.471489 |  |
| pct_sf3_h019093 | 0.084937 | 1.088648 | 0.136616 | 0.621721 | 0.534125 | 0.83291 | 1.422909 |  |
| pct_sf3_h045025 | 0.150232 | 1.162103 | 0.128788 | 1.166504 | 0.243411 | 0.902856 | 1.495792 |  |
| pct_sf3_pct050102 | 0.112489 | 1.11906 | 0.132921 | 0.846284 | 0.397395 | 0.8624 | 1.452104 |  |
| pct_sf3_pct051020 | 0.059572 | 1.061382 | 0.135011 | 0.441237 | 0.659041 | 0.814606 | 1.382916 |  |
| pct_sf3_pct075a006 | 0.004747 | 1.004759 | 0.148869 | 0.03189 | 0.97456 | 0.750485 | 1.345183 |  |
| pct_sf3_hct004093 | 0.277974 | 1.320452 | 0.124336 | 2.235668 | 0.025374 | 1.03487 | 1.684843 | * (LASSO) |
| pct_sf3_hct005083 | 0.029814 | 1.030263 | 0.132523 | 0.224974 | 0.821999 | 0.794588 | 1.33584 |  |
| pct_sf3_hct015042 | 0.163504 | 1.17763 | 0.126503 | 1.292487 | 0.196189 | 0.919024 | 1.509005 |  |
| pct_sf3_hct017019 | -0.0126 | 0.98748 | 0.140953 | -0.08939 | 0.928775 | 0.749112 | 1.301697 |  |
| pct_sf1_p030012 | 0.117594 | 1.124787 | 0.137883 | 0.852854 | 0.39374 | 0.858425 | 1.4738 |  |
| PCT_SF3_H021045 | 0.266391 | 1.305245 | 0.127698 | 2.086091 | 0.03697 | 1.016232 | 1.676452 | * |
| PCT_SF3_H111002 | 0.085088 | 1.088813 | 0.129738 | 0.655849 | 0.511921 | 0.844342 | 1.404069 |  |
| PCT_SF3_PCT025051 | 0.057642 | 1.059336 | 0.124941 | 0.461351 | 0.644547 | 0.829242 | 1.353274 |  |
| PCT_SF3_P033003 | 0.123087 | 1.130983 | 0.131798 | 0.933911 | 0.35035 | 0.873509 | 1.464349 |  |
| PCT_SF3_H042006 | 0.281915 | 1.325666 | 0.135098 | 2.086744 | 0.036911 | 1.01727 | 1.727554 | * (LASSO) |
| PCT_SF3_P010015 | 0.255837 | 1.291542 | 0.134535 | 1.901634 | 0.057219 | 0.992178 | 1.681231 | * |
| PCT_SE_T059_002 | -0.07941 | 0.923665 | 0.137803 | -0.57623 | 0.564461 | 0.705041 | 1.210081 |  |
| popzeronine | -0.00134 | 0.998663 | 0.131348 | -0.01019 | 0.99187 | 0.771993 | 1.291886 |  |
| poptennineteen | 0.050338 | 1.051627 | 0.137157 | 0.36701 | 0.713612 | 0.803731 | 1.375981 |  |
| SF3_P053001 | -0.12079 | 0.886218 | 0.136622 | -0.88414 | 0.376623 | 0.678026 | 1.158338 |  |
| SF3_H085001 | -0.17302 | 0.84112 | 0.132197 | -1.30881 | 0.190597 | 0.649127 | 1.089899 |  |
| novehicle | 0.145419 | 1.156525 | 0.136294 | 1.066956 | 0.285992 | 0.8854 | 1.510673 |  |
| PCT_SF3_P087002 | 0.161048 | 1.174741 | 0.13114 | 1.228059 | 0.219425 | 0.908476 | 1.519047 |  |
| PCT_SF1_P007003 | 0.223788 | 1.250806 | 0.128196 | 1.745676 | 0.080867 | 0.972899 | 1.608098 | * |
| PCT_SF1_P007002 | -0.24589 | 0.782009 | 0.130834 | -1.87939 | 0.060191 | 0.605122 | 1.010602 | * |
| PCT_SF1_P010009 | 0.083982 | 1.08761 | 0.136096 | 0.617084 | 0.537179 | 0.832964 | 1.420103 |  |
| PCT_SF1_P007007 | 0.074249 | 1.077075 | 0.135396 | 0.548385 | 0.583427 | 0.826027 | 1.404422 |  |
| PCT_SE_T069_006_y | 0.18443 | 1.202533 | 0.135839 | 1.35771 | 0.174556 | 0.921443 | 1.569372 |  |
| collegeover25_y | -0.17017 | 0.84352 | 0.138824 | -1.22581 | 0.22027 | 0.642579 | 1.107298 |  |
| PCT_SE_T040_002_y | 0.072447 | 1.075136 | 0.133809 | 0.541423 | 0.588216 | 0.827109 | 1.397541 |  |
| PCT_SE_T085_017 | -0.05505 | 0.946435 | 0.138401 | -0.39778 | 0.690794 | 0.721575 | 1.241367 |  |
| PCT_SF3_P018013 | -0.14792 | 0.862498 | 0.125523 | -1.17844 | 0.23862 | 0.674389 | 1.103077 |  |
| PCT_SF3_P021013 | 0.095908 | 1.100658 | 0.136005 | 0.705179 | 0.480699 | 0.843107 | 1.436885 |  |
| SF1_P037002 | 0.019088 | 1.019271 | 0.132543 | 0.144014 | 0.885489 | 0.786081 | 1.321638 |  |
| houses1970 | -0.29765 | 0.74256 | 0.15132 | -1.96703 | 0.04918 | 0.551982 | 0.998937 | * |
| PCT_SF3_H034009 | 0.135206 | 1.144773 | 0.124881 | 1.082679 | 0.278951 | 0.896228 | 1.462246 |  |
| PCT_SF3_H038002 | -0.07352 | 0.929117 | 0.128659 | -0.57144 | 0.567701 | 0.722028 | 1.195602 |  |
| PCT_SF3_H047003 | 0.065919 | 1.06814 | 0.132622 | 0.497042 | 0.619159 | 0.823641 | 1.385217 |  |
| PCT_SF3_P115007 | 0.172935 | 1.188789 | 0.142431 | 1.214172 | 0.224682 | 0.899219 | 1.571608 |  |
| PCT_SE_T159_002 | 0.043844 | 1.044819 | 0.128651 | 0.3408 | 0.733254 | 0.811955 | 1.344469 |  |
| vacanthouse | -0.03345 | 0.967099 | 0.142685 | -0.23446 | 0.814625 | 0.731164 | 1.279166 |  |
| PCT_SE_T027_002 | 0.124454 | 1.13253 | 0.112811 | 1.103202 | 0.269939 | 0.907868 | 1.412787 |  |
| PCT_SE_T027_003 | -0.1786 | 0.836442 | 0.127891 | -1.39649 | 0.162567 | 0.650988 | 1.074728 |  |
| hscollegegrad | -0.12552 | 0.882038 | 0.134035 | -0.93647 | 0.34903 | 0.678258 | 1.147044 |  |
| morecollege | -0.11738 | 0.889246 | 0.132442 | -0.88628 | 0.375466 | 0.685939 | 1.152813 |  |
| PCT_SE_T069_005 | -0.13211 | 0.876245 | 0.132195 | -0.99936 | 0.317623 | 0.676237 | 1.135408 |  |
| SE_T070_002 | -0.19775 | 0.820571 | 0.146346 | -1.35128 | 0.176606 | 0.615948 | 1.093172 |  |
| PCT_SE_T070_006 | 0.220677 | 1.246921 | 0.137978 | 1.599365 | 0.109739 | 0.951458 | 1.634136 |  |
| workingclass | 0.137939 | 1.147905 | 0.135378 | 1.018914 | 0.308244 | 0.880379 | 1.496726 |  |
| PCT_SE_T086_002 | -0.14749 | 0.862868 | 0.137624 | -1.07171 | 0.28385 | 0.658865 | 1.130037 |  |
| PCT_SE_T086_003 | -0.10049 | 0.904398 | 0.136561 | -0.73583 | 0.461835 | 0.692016 | 1.18196 |  |
| bluecollar | 0.006402 | 1.006423 | 0.136634 | 0.046858 | 0.962627 | 0.769973 | 1.315485 | LASSO |
| PCT_SE_T086_012 | 0.134594 | 1.144072 | 0.129706 | 1.037685 | 0.299417 | 0.887249 | 1.475236 |  |
| PCT_SF1_P020012 | 0.272665 | 1.313461 | 0.133843 | 2.037197 | 0.04163 | 1.010386 | 1.707446 | * |
| PCT_SF1_P010010 | 0.013236 | 1.013324 | 0.146417 | 0.090396 | 0.927972 | 0.760528 | 1.350147 |  |
| PCT_SF1_P010011 | 0.114687 | 1.121522 | 0.130492 | 0.878878 | 0.379468 | 0.868422 | 1.448387 |  |
| PCT_SF1_P012B002 | -0.38356 | 0.68143 | 0.156695 | -2.44782 | 0.014372 | 0.501232 | 0.926409 | * (LASSO) |
| PCT_SF1_H003003 | -0.01771 | 0.982449 | 0.144258 | -0.12275 | 0.902308 | 0.740482 | 1.303482 |  |
| PCT_SF3_P064002 | 0.161128 | 1.174836 | 0.132674 | 1.214467 | 0.224569 | 0.905822 | 1.523743 |  |
| hhincome15k | 0.093934 | 1.098488 | 0.134651 | 0.697612 | 0.48542 | 0.84368 | 1.430252 |  |
| hhincome150k | -0.30935 | 0.733921 | 0.145378 | -2.12792 | 0.033343 | 0.551952 | 0.975883 |  |
| PCT_SF3_P052002 | 0.097614 | 1.102537 | 0.135112 | 0.722468 | 0.470007 | 0.846026 | 1.436821 |  |
| PCT_SF3_P052003 | -0.00284 | 0.997165 | 0.131022 | -0.02166 | 0.982715 | 0.771327 | 1.289127 |  |
| SF3_P077001 | -0.17372 | 0.840532 | 0.135851 | -1.27876 | 0.200981 | 0.644044 | 1.096965 |  |
| PCT_SF3_P089002 | 0.161048 | 1.174741 | 0.13114 | 1.228059 | 0.219425 | 0.908476 | 1.519047 |  |
| PCT_SF3_P089021 | -0.0776 | 0.925331 | 0.115753 | -0.67042 | 0.502587 | 0.737507 | 1.160989 |  |
| PCT_SF3_P090002 | 0.171756 | 1.187388 | 0.132251 | 1.298714 | 0.194042 | 0.916259 | 1.538746 |  |
| PCT_SF3_P092002 | 0.134509 | 1.143976 | 0.132967 | 1.011601 | 0.311729 | 0.881522 | 1.484569 |  |
| PCT_SF3_H020002 | -0.07352 | 0.929117 | 0.128659 | -0.57144 | 0.567701 | 0.722028 | 1.195602 |  |
| crowding | -0.01247 | 0.987604 | 0.134664 | -0.09263 | 0.926199 | 0.758498 | 1.285911 |  |
| homeprice | -0.26464 | 0.767483 | 0.145608 | -1.81749 | 0.069143 | 0.576932 | 1.020968 | * |

1. **White Men Only**

|  | coef | HR | se(coef) | z | Pval | 95% CI |  | P-value <0.10; tried in multivariate model (also found in Lasso) |
| --- | --- | --- | --- | --- | --- | --- | --- | --- |
| pct_sf3_p030007 | 0.881326 | 2.414098 | 0.251488 | 3.504442 | 0.000458 | 1.474633 | 3.952082 | * (LASSO) |
| pct_sf3_p050026 | 0.208819 | 1.232222 | 0.234452 | 0.890668 | 0.373107 | 0.77825 | 1.951008 |  |
| pct_sf3_p052012 | 0.272502 | 1.313246 | 0.293874 | 0.927275 | 0.353784 | 0.738236 | 2.336128 |  |
| pct_sf3_p084006 | 0.66901 | 1.952304 | 0.28029 | 2.386853 | 0.016993 | 1.127094 | 3.381698 | * (LASSO) |
| pct_sf3_p092021 | -0.21167 | 0.809233 | 0.330977 | -0.63953 | 0.52248 | 0.422999 | 1.548129 |  |
| pct_sf3_p120002 | 0.014071 | 1.014171 | 0.33711 | 0.041741 | 0.966705 | 0.52379 | 1.963653 |  |
| pct_sf3_p159i007 | -0.1325 | 0.875899 | 0.413546 | -0.32041 | 0.748658 | 0.389436 | 1.970026 |  |
| pct_sf3_h019093 | -0.0124 | 0.987672 | 0.233569 | -0.05311 | 0.957645 | 0.624877 | 1.561101 |  |
| pct_sf3_h045025 | 0.119659 | 1.127112 | 0.432329 | 0.276777 | 0.781951 | 0.483016 | 2.630102 |  |
| pct_sf3_pct050102 | -0.27539 | 0.759275 | 0.521526 | -0.52805 | 0.597464 | 0.273191 | 2.110233 | LASSO |
| pct_sf3_pct051020 | 0.057963 | 1.059676 | 0.242302 | 0.239218 | 0.810937 | 0.659054 | 1.703825 |  |
| pct_sf3_pct075a006 | 0.668325 | 1.950966 | 0.337053 | 1.982848 | 0.047384 | 1.007731 | 3.777069 | * (LASSO) |
| pct_sf3_hct004093 | 0.61802 | 1.855251 | 0.225875 | 2.736116 | 0.006217 | 1.19161 | 2.888494 | * (LASSO) |
| pct_sf3_hct005083 | -0.4421 | 0.642686 | 0.381259 | -1.15958 | 0.246221 | 0.304414 | 1.356854 | LASSO |
| pct_sf3_hct015042 | -0.01427 | 0.985832 | 0.310942 | -0.04589 | 0.963398 | 0.53595 | 1.813352 |  |
| pct_sf3_hct017019 | 0.195364 | 1.215754 | 0.474331 | 0.411874 | 0.680432 | 0.47983 | 3.080378 |  |
| pct_sf1_p030012 | 0.215344 | 1.240288 | 0.244799 | 0.879676 | 0.379035 | 0.767619 | 2.004009 |  |
| PCT_SF3_H021045 | 0.492456 | 1.63633 | 0.311517 | 1.580831 | 0.103917 | 0.888592 | 3.013279 | * (LASSO) |
| PCT_SF3_H111002 | 0.051055 | 1.052381 | 0.299229 | 0.170623 | 0.86452 | 0.585416 | 1.891828 |  |
| PCT_SF3_P033003 | 0.043965 | 1.044946 | 0.303035 | 0.145084 | 0.884645 | 0.57696 | 1.892529 |  |
| PCT_SF3_H042006 | 0.545232 | 1.725008 | 0.256736 | 2.123708 | 0.033695 | 1.042926 | 2.853179 | * |
| PCT_SF3_P010015 | 0.667229 | 1.94883 | 0.351425 | 1.898637 | 0.057612 | 0.978666 | 3.880728 | * |
| PCT_SE_T059_002 | -0.31805 | 0.727569 | 0.281555 | -1.1296 | 0.258643 | 0.418996 | 1.263395 | LASSO |
| popzeronine | 0.195041 | 1.215361 | 0.219514 | 0.888514 | 0.374264 | 0.790408 | 1.868784 |  |
| poptennineteen | -0.12043 | 0.886537 | 0.27215 | -0.44252 | 0.658111 | 0.520042 | 1.511317 | LASSO |
| SF3_P053001 | -0.09015 | 0.913799 | 0.25047 | -0.3599 | 0.718918 | 0.559302 | 1.492982 |  |
| SF3_H085001 | -0.49704 | 0.608329 | 0.328008 | -1.51533 | 0.129689 | 0.31984 | 1.157029 |  |
| novehicle | 0.244592 | 1.2771 | 0.291293 | 0.839677 | 0.401089 | 0.721559 | 2.260362 |  |
| PCT_SF3_P087002 | 0.034904 | 1.03552 | 0.327341 | 0.106628 | 0.915084 | 0.545155 | 1.966966 |  |
| PCT_SF1_P007003 | -0.35031 | 0.70447 | 1.378785 | -0.25407 | 0.799441 | 0.04723 | 10.50772 |  |
| PCT_SF1_P007002 | 0.203853 | 1.226118 | 0.972379 | 0.209644 | 0.833946 | 0.182317 | 8.245895 |  |
| PCT_SF1_P010009 | 0.243517 | 1.275728 | 0.276631 | 0.880297 | 0.378699 | 0.741797 | 2.193971 |  |
| PCT_SF1_P007007 | 0.143305 | 1.154082 | 0.318042 | 0.450587 | 0.652288 | 0.618749 | 2.152579 |  |
| PCT_SE_T069_006_y | 0.023007 | 1.023274 | 0.3706 | 0.06208 | 0.950499 | 0.494915 | 2.115694 |  |
| collegeover25_y | -0.31564 | 0.72932 | 0.263616 | -1.19736 | 0.231166 | 0.435034 | 1.222679 |  |
| PCT_SE_T040_002_y | 0.169248 | 1.184414 | 0.308412 | 0.548774 | 0.583161 | 0.64711 | 2.16785 |  |
| PCT_SE_T085_017 | 0.050501 | 1.051798 | 0.261581 | 0.193059 | 0.846913 | 0.629897 | 1.756284 |  |
| PCT_SF3_P018013 | -0.19949 | 0.819147 | 0.237898 | -0.83856 | 0.401717 | 0.513877 | 1.305765 |  |
| PCT_SF3_P021013 | 0.079066 | 1.082276 | 0.241386 | 0.32755 | 0.743252 | 0.67432 | 1.73704 |  |
| SF1_P037002 | -0.12201 | 0.88514 | 0.258144 | -0.47264 | 0.636469 | 0.533673 | 1.468076 |  |
| houses1970 | -0.30736 | 0.735383 | 0.254993 | -1.20538 | 0.228055 | 0.446128 | 1.212181 |  |
| PCT_SF3_H034009 | 0.061402 | 1.063326 | 0.249577 | 0.246025 | 0.805663 | 0.651962 | 1.734246 |  |
| PCT_SF3_H038002 | -0.01852 | 0.98165 | 0.255914 | -0.07237 | 0.942308 | 0.594455 | 1.621044 |  |
| PCT_SF3_H047003 | 0.004574 | 1.004584 | 0.441074 | 0.01037 | 0.991726 | 0.423191 | 2.384715 |  |
| PCT_SF3_P115007 | 0.247185 | 1.280416 | 0.276705 | 0.893317 | 0.371688 | 0.744415 | 2.202352 |  |
| PCT_SE_T159_002 | 0.19584 | 1.216332 | 0.259757 | 0.753933 | 0.450889 | 0.731042 | 2.023772 |  |
| vacanthouse | 0.145914 | 1.157096 | 0.340504 | 0.428523 | 0.668271 | 0.593645 | 2.255341 |  |
| PCT_SE_T027_002 | 0.042047 | 1.042943 | 0.22494 | 0.186925 | 0.85172 | 0.671101 | 1.620815 |  |
| PCT_SE_T027_003 | -0.23114 | 0.793628 | 0.258967 | -0.89255 | 0.3721 | 0.477727 | 1.318422 |  |
| hscollegegrad | -0.15326 | 0.85791 | 0.334457 | -0.45822 | 0.646791 | 0.445396 | 1.652483 |  |
| morecollege | -0.41228 | 0.662138 | 0.26577 | -1.55128 | 0.120836 | 0.393297 | 1.114746 |  |
| PCT_SE_T069_005 | 0.205649 | 1.228322 | 0.349083 | 0.589111 | 0.555787 | 0.619679 | 2.434768 |  |
| SE_T070_002 | 0.119872 | 1.127352 | 0.229829 | 0.521568 | 0.601971 | 0.718497 | 1.768863 |  |
| PCT_SE_T070_006 | 0.176991 | 1.193621 | 0.352552 | 0.502029 | 0.615647 | 0.598092 | 2.382127 |  |
| workingclass | 0.300663 | 1.350754 | 0.267846 | 1.122521 | 0.261641 | 0.799063 | 2.283344 |  |
| PCT_SE_T086_002 | -0.14528 | 0.864776 | 0.239766 | -0.60594 | 0.544552 | 0.540519 | 1.383555 |  |
| PCT_SE_T086_003 | -0.32524 | 0.722357 | 0.283555 | -1.14699 | 0.251385 | 0.414367 | 1.259271 |  |
| bluecollar | 0.110216 | 1.11652 | 0.202891 | 0.54323 | 0.586972 | 0.750174 | 1.661769 |  |
| PCT_SE_T086_012 | 0.34861 | 1.417096 | 0.207444 | 1.680502 | 0.09286 | 0.943669 | 2.128036 | * |
| PCT_SF1_P020012 | 0.749971 | 2.116939 | 0.368182 | 2.03696 | 0.041654 | 1.02874 | 4.356231 | * |
| PCT_SF1_P010010 | 0.278808 | 1.321553 | 0.261482 | 1.06626 | 0.286306 | 0.791602 | 2.206291 |  |
| PCT_SF1_P010011 | -0.51756 | 0.595974 | 0.700837 | -0.73849 | 0.460219 | 0.150891 | 2.353917 |  |
| PCT_SF1_P012B002 | -0.22974 | 0.794736 | 0.212626 | -1.08051 | 0.279915 | 0.52388 | 1.20563 |  |
| PCT_SF1_H003003 | 0.082482 | 1.085979 | 0.358854 | 0.229848 | 0.81821 | 0.537475 | 2.194241 |  |
| PCT_SF3_P064002 | 0.662131 | 1.938919 | 0.40661 | 1.628417 | 0.103436 | 0.873869 | 4.302024 | * |
| hhincome15k | -0.17754 | 0.837325 | 0.300375 | -0.59107 | 0.554471 | 0.464739 | 1.508614 | LASSO |
| hhincome150k | -0.26276 | 0.768925 | 0.261886 | -1.00335 | 0.315693 | 0.460216 | 1.284711 |  |
| PCT_SF3_P052002 | -0.08562 | 0.91794 | 0.32365 | -0.26456 | 0.791352 | 0.486763 | 1.731054 |  |
| PCT_SF3_P052003 | -0.05355 | 0.947859 | 0.294684 | -0.18172 | 0.855805 | 0.53199 | 1.688823 |  |
| SF3_P077001 | -0.13773 | 0.87133 | 0.266335 | -0.51715 | 0.605054 | 0.51698 | 1.468561 |  |
| PCT_SF3_P089002 | 0.034904 | 1.03552 | 0.327341 | 0.106628 | 0.915084 | 0.545155 | 1.966966 |  |
| PCT_SF3_P089021 | 0.109264 | 1.115457 | 0.371886 | 0.293811 | 0.768902 | 0.538142 | 2.312112 |  |
| PCT_SF3_P090002 | 0.103025 | 1.108519 | 0.333313 | 0.309095 | 0.757249 | 0.576795 | 2.130418 |  |
| PCT_SF3_P092002 | 0.004193 | 1.004202 | 0.328077 | 0.01278 | 0.989804 | 0.527906 | 1.910229 |  |
| PCT_SF3_H020002 | -0.01852 | 0.98165 | 0.255914 | -0.07237 | 0.942308 | 0.594455 | 1.621044 |  |
| crowding | 0.031346 | 1.031842 | 0.270176 | 0.11602 | 0.907637 | 0.607624 | 1.752232 | LASSO |
| homeprice | -0.4526 | 0.635971 | 0.247233 | -1.83067 | 0.06715 | 0.391731 | 1.032492 | * (LASSO) |

1. **Black Men without a Prostate Cancer Family History**

|  | coef | HR | se(coef) | z | Pval | 95% CI |  | P-value <0.10*; tried in multivariate model (also found in Lasso) |
| --- | --- | --- | --- | --- | --- | --- | --- | --- |
| pct_sf3_p052012 | -0.25798 | 0.772608 | 0.295696 | -0.87246 | 0.382957 | 0.43277 | 1.379307 |  |
| pct_sf3_h019093 | 0.089583 | 1.093718 | 0.272068 | 0.329265 | 0.741955 | 0.641677 | 1.864207 |  |
| pct_sf3_pct051020 | 0.474933 | 1.607906 | 0.279996 | 1.696212 | 0.089846 | 0.928802 | 2.783544 | * |
| pct_sf3_hct005083 | 0.282476 | 1.32641 | 0.217534 | 1.298541 | 0.194101 | 0.865983 | 2.031637 |  |
| pct_sf1_p030012 | -0.10572 | 0.899674 | 0.233708 | -0.45237 | 0.651 | 0.569048 | 1.422398 |  |
| PCT_SF3_H021045 | 0.139663 | 1.149886 | 0.2732 | 0.511211 | 0.609203 | 0.673135 | 1.964298 |  |
| PCT_SF3_H111002 | -0.24553 | 0.782289 | 0.289057 | -0.84942 | 0.395647 | 0.443932 | 1.378535 |  |
| PCT_SF3_PCT025051 | 0.213993 | 1.238614 | 0.179935 | 1.18928 | 0.23433 | 0.870507 | 1.76238 |  |
| PCT_SF3_P033003 | -0.0375 | 0.963194 | 0.275984 | -0.13588 | 0.891916 | 0.560778 | 1.654384 |  |
| PCT_SF3_H042006 | 0.162804 | 1.176806 | 0.242203 | 0.672179 | 0.50147 | 0.732044 | 1.891786 |  |
| PCT_SF3_P010015 | 0.261869 | 1.299356 | 0.316169 | 0.828254 | 0.407526 | 0.699197 | 2.414665 |  |
| PCT_SE_T059_002 | -0.17798 | 0.836962 | 0.264397 | -0.67314 | 0.500858 | 0.498478 | 1.405288 |  |
| popzeronine | 0.147167 | 1.158548 | 0.208537 | 0.705713 | 0.480366 | 0.769846 | 1.743509 |  |
| poptennineteen | 0.37133 | 1.449661 | 0.233651 | 1.589252 | 0.092003 | 0.91702 | 2.291678 | * (LASSO) |
| SF3_P053001 | 0.130817 | 1.139759 | 0.336965 | 0.388221 | 0.697853 | 0.58882 | 2.206194 |  |
| SF3_H085001 | -0.05425 | 0.947194 | 0.316782 | -0.17126 | 0.864021 | 0.509083 | 1.76234 |  |
| novehicle | 0.069002 | 1.071439 | 0.310426 | 0.222283 | 0.824094 | 0.583079 | 1.968826 |  |
| PCT_SF3_P087002 | 0.304919 | 1.356516 | 0.305143 | 0.999268 | 0.317665 | 0.745902 | 2.466991 |  |
| PCT_SF1_P007003 | 0.129034 | 1.137729 | 0.284176 | 0.454063 | 0.649783 | 0.651843 | 1.985794 |  |
| PCT_SF1_P007002 | -0.26127 | 0.770071 | 0.31418 | -0.8316 | 0.405635 | 0.416002 | 1.425498 |  |
| PCT_SF1_P010009 | 0.104949 | 1.110654 | 0.249379 | 0.420842 | 0.67387 | 0.681245 | 1.810731 |  |
| PCT_SF1_P007007 | 0.119917 | 1.127403 | 0.2416 | 0.496344 | 0.619652 | 0.702142 | 1.810227 |  |
| PCT_SE_T069_006_y | 0.446574 | 1.562949 | 0.257155 | 1.736596 | 0.082458 | 0.94417 | 2.587257 | * (LASSO) |
| collegeover25_y | -0.07722 | 0.92569 | 0.297253 | -0.25977 | 0.795044 | 0.516938 | 1.657646 |  |
| PCT_SE_T040_002_y | 0.184426 | 1.202528 | 0.289988 | 0.635979 | 0.52479 | 0.681165 | 2.122943 |  |
| PCT_SE_T085_017 | 0.160315 | 1.17388 | 0.249352 | 0.642926 | 0.520272 | 0.720064 | 1.913712 |  |
| PCT_SF3_P018013 | 0.123725 | 1.131705 | 0.246467 | 0.501996 | 0.615671 | 0.69813 | 1.834554 |  |
| PCT_SF3_P021013 | 0.324345 | 1.383124 | 0.302902 | 1.070789 | 0.284264 | 0.76388 | 2.50436 |  |
| SF1_P037002 | 0.002774 | 1.002777 | 0.27305 | 0.010158 | 0.991896 | 0.587191 | 1.712497 |  |
| houses1970 | -0.26513 | 0.767109 | 0.3588 | -0.73893 | 0.459951 | 0.3797 | 1.549791 |  |
| PCT_SF3_H034009 | 0.048273 | 1.049458 | 0.248101 | 0.194572 | 0.845728 | 0.645323 | 1.706681 |  |
| PCT_SF3_H038002 | 0.011162 | 1.011225 | 0.241426 | 0.046234 | 0.963124 | 0.630001 | 1.623132 |  |
| PCT_SF3_H047003 | 0.217385 | 1.242822 | 0.225843 | 0.96255 | 0.335773 | 0.798303 | 1.934863 |  |
| PCT_SF3_P115007 | 0.093458 | 1.097964 | 0.236759 | 0.394738 | 0.693037 | 0.690327 | 1.746311 |  |
| PCT_SE_T159_002 | 0.145096 | 1.15615 | 0.223499 | 0.649203 | 0.516207 | 0.746051 | 1.791679 |  |
| vacanthouse | -0.40537 | 0.66673 | 0.309833 | -1.30835 | 0.190755 | 0.363258 | 1.22373 |  |
| PCT_SE_T027_002 | 0.00764 | 1.007669 | 0.305877 | 0.024976 | 0.980074 | 0.553287 | 1.835208 |  |
| PCT_SE_T027_003 | 0.079308 | 1.082537 | 0.283115 | 0.280125 | 0.779381 | 0.621513 | 1.885538 |  |
| hscollegegrad | -0.19643 | 0.821656 | 0.301123 | -0.65233 | 0.514185 | 0.455375 | 1.482557 |  |
| morecollege | 0.025752 | 1.026086 | 0.268854 | 0.095783 | 0.923693 | 0.605802 | 1.737949 |  |
| PCT_SE_T069_005 | -0.48983 | 0.612729 | 0.284234 | -1.72335 | 0.084826 | 0.351013 | 1.069578 |  |
| SE_T070_002 | -0.41306 | 0.661621 | 0.324794 | -1.27177 | 0.203456 | 0.350057 | 1.250488 |  |
| PCT_SE_T070_006 | 0.419456 | 1.521133 | 0.263017 | 1.594787 | 0.11076 | 0.908412 | 2.547133 |  |
| workingclass | 0.110917 | 1.117303 | 0.27395 | 0.404883 | 0.685564 | 0.653101 | 1.911443 |  |
| PCT_SE_T086_002 | -0.03604 | 0.964604 | 0.321521 | -0.11208 | 0.910756 | 0.513648 | 1.811477 |  |
| PCT_SE_T086_003 | -0.12493 | 0.882558 | 0.256285 | -0.48747 | 0.625927 | 0.534059 | 1.45847 |  |
| bluecollar | 0.012709 | 1.01279 | 0.302554 | 0.042004 | 0.966495 | 0.559732 | 1.83256 |  |
| PCT_SE_T086_012 | -0.15118 | 0.859697 | 0.307532 | -0.49158 | 0.623019 | 0.47051 | 1.570803 |  |
| PCT_SF1_P020012 | 0.243091 | 1.275185 | 0.296975 | 0.818558 | 0.413039 | 0.712497 | 2.282248 |  |
| PCT_SF1_P010010 | 0.081643 | 1.085069 | 0.246375 | 0.331378 | 0.740359 | 0.669481 | 1.758636 |  |
| PCT_SF1_P010011 | -0.07729 | 0.925621 | 0.294157 | -0.26275 | 0.792741 | 0.520046 | 1.647497 |  |
| PCT_SF1_P012B002 | -0.18865 | 0.828076 | 0.462993 | -0.40746 | 0.683672 | 0.334166 | 2.052004 |  |
| PCT_SF1_H003003 | -0.30388 | 0.737948 | 0.296052 | -1.02645 | 0.304681 | 0.413067 | 1.31835 |  |
| PCT_SF3_P064002 | 0.272637 | 1.313424 | 0.281758 | 0.967627 | 0.333231 | 0.756079 | 2.281615 |  |
| hhincome15k | 0.074472 | 1.077316 | 0.317015 | 0.234917 | 0.814273 | 0.578755 | 2.005355 |  |
| hhincome150k | -0.236 | 0.789777 | 0.35373 | -0.66719 | 0.504653 | 0.394824 | 1.579814 |  |
| PCT_SF3_P052002 | 0.05634 | 1.057958 | 0.302136 | 0.186473 | 0.852074 | 0.585174 | 1.912721 |  |
| PCT_SF3_P052003 | -0.01229 | 0.987787 | 0.279122 | -0.04402 | 0.964886 | 0.571571 | 1.707092 |  |
| SF3_P077001 | -0.15656 | 0.855083 | 0.340309 | -0.46004 | 0.645485 | 0.438865 | 1.666039 |  |
| PCT_SF3_P089002 | 0.304919 | 1.356516 | 0.305143 | 0.999268 | 0.317665 | 0.745902 | 2.466991 |  |
| PCT_SF3_P089021 | -0.26937 | 0.76386 | 0.245219 | -1.09849 | 0.271991 | 0.472366 | 1.235232 |  |
| PCT_SF3_P090002 | 0.435053 | 1.545045 | 0.328213 | 1.32552 | 0.184999 | 0.812008 | 2.939828 |  |
| PCT_SF3_P092002 | 0.177348 | 1.194046 | 0.301192 | 0.58882 | 0.555982 | 0.66167 | 2.154771 |  |
| PCT_SF3_H020002 | 0.011162 | 1.011225 | 0.241426 | 0.046234 | 0.963124 | 0.630001 | 1.623132 |  |
| crowding | -0.01466 | 0.98545 | 0.259591 | -0.05646 | 0.954973 | 0.59247 | 1.63909 |  |
| homeprice | 0.143057 | 1.153796 | 0.313599 | 0.456179 | 0.648261 | 0.624005 | 2.133388 |  |

1. **Black Men with a Prostate Cancer Family History**

|  | coef | HR | se(coef) | z | Pval | 95% CI |  | P-value <0.10; tried in multivariate model (also found in Lasso) |
| --- | --- | --- | --- | --- | --- | --- | --- | --- |
| pct_sf3_p052012 | 0.34561 | 1.412852 | 0.243814 | 1.417513 | 0.156333 | 0.876108 | 2.278429 |  |
| pct_sf3_h019093 | -0.19772 | 0.820598 | 0.22963 | -0.86105 | 0.389213 | 0.523197 | 1.28705 |  |
| pct_sf3_pct051020 | -0.14882 | 0.861728 | 0.20529 | -0.72491 | 0.46851 | 0.576267 | 1.288594 |  |
| pct_sf3_hct005083 | -0.14235 | 0.867322 | 0.238199 | -0.59759 | 0.550113 | 0.543777 | 1.383372 |  |
| pct_sf1_p030012 | 0.074878 | 1.077753 | 0.217499 | 0.344269 | 0.730644 | 0.703688 | 1.650662 |  |
| PCT_SF3_H021045 | 0.040936 | 1.041786 | 0.201523 | 0.203133 | 0.839031 | 0.70184 | 1.546387 |  |
| PCT_SF3_H111002 | 0.024375 | 1.024674 | 0.198875 | 0.122563 | 0.902453 | 0.693906 | 1.513113 |  |
| PCT_SF3_PCT025051 | -0.00408 | 0.995927 | 0.266322 | -0.01532 | 0.987773 | 0.590922 | 1.678515 |  |
| PCT_SF3_P033003 | -0.07249 | 0.930072 | 0.239287 | -0.30295 | 0.761924 | 0.581878 | 1.486626 |  |
| PCT_SF3_H042006 | 0.365249 | 1.440873 | 0.20335 | 1.796162 | 0.072469 | 0.967233 | 2.146447 | * (LASSO) |
| PCT_SF3_P010015 | -0.06262 | 0.939301 | 0.224893 | -0.27844 | 0.780675 | 0.604466 | 1.459614 |  |
| PCT_SE_T059_002 | 0.086999 | 1.090895 | 0.207667 | 0.418935 | 0.675264 | 0.726129 | 1.638901 |  |
| popzeronine | -0.31572 | 0.729267 | 0.254947 | -1.23835 | 0.215585 | 0.442457 | 1.201994 |  |
| poptennineteen | -0.13676 | 0.872179 | 0.22702 | -0.60242 | 0.546896 | 0.558936 | 1.36097 |  |
| SF3_P053001 | 0.033674 | 1.034247 | 0.213553 | 0.157684 | 0.874706 | 0.680526 | 1.571824 |  |
| SF3_H085001 | 0.081846 | 1.085289 | 0.196153 | 0.417258 | 0.67649 | 0.738885 | 1.594094 |  |
| novehicle | -0.16541 | 0.847546 | 0.206139 | -0.80242 | 0.422308 | 0.565841 | 1.269498 |  |
| PCT_SF3_P087002 | -0.12249 | 0.884716 | 0.205023 | -0.59744 | 0.550214 | 0.59195 | 1.322278 |  |
| PCT_SF1_P007003 | 0.130797 | 1.139736 | 0.213578 | 0.612409 | 0.540268 | 0.7499 | 1.732229 |  |
| PCT_SF1_P007002 | -0.07932 | 0.923749 | 0.220008 | -0.36051 | 0.718466 | 0.600177 | 1.421767 |  |
| PCT_SF1_P010009 | -0.24962 | 0.779097 | 0.245366 | -1.01734 | 0.308993 | 0.481651 | 1.260233 |  |
| PCT_SF1_P007007 | -0.1838 | 0.832102 | 0.22796 | -0.80628 | 0.420081 | 0.532271 | 1.30083 |  |
| PCT_SE_T069_006_y | -0.2132 | 0.807994 | 0.238767 | -0.89292 | 0.371897 | 0.506017 | 1.290181 |  |
| collegeover25_y | -0.01139 | 0.988677 | 0.218049 | -0.05222 | 0.958351 | 0.644834 | 1.515869 |  |
| PCT_SE_T040_002_y | -0.2027 | 0.816524 | 0.207522 | -0.97676 | 0.328687 | 0.543654 | 1.226351 |  |
| PCT_SE_T085_017 | -0.12064 | 0.886352 | 0.242622 | -0.49724 | 0.619019 | 0.550911 | 1.426035 |  |
| PCT_SF3_P018013 | -0.00958 | 0.990468 | 0.218492 | -0.04384 | 0.965036 | 0.645441 | 1.519933 |  |
| PCT_SF3_P021013 | -0.10352 | 0.901662 | 0.207856 | -0.49801 | 0.618474 | 0.599948 | 1.355109 |  |
| SF1_P037002 | -0.10182 | 0.90319 | 0.255591 | -0.39838 | 0.690351 | 0.547287 | 1.490538 |  |
| houses1970 | -0.04146 | 0.95939 | 0.251714 | -0.1647 | 0.869178 | 0.585776 | 1.571298 |  |
| PCT_SF3_H034009 | 0.29929 | 1.348901 | 0.28236 | 1.05996 | 0.289163 | 0.775587 | 2.346008 |  |
| PCT_SF3_H038002 | 0.218005 | 1.243593 | 0.236219 | 0.922892 | 0.356063 | 0.782716 | 1.975842 |  |
| PCT_SF3_H047003 | -0.22068 | 0.801975 | 0.21606 | -1.02138 | 0.307076 | 0.525106 | 1.224826 |  |
| PCT_SF3_P115007 | 0.372243 | 1.450985 | 0.252951 | 1.471602 | 0.141128 | 0.883786 | 2.382203 |  |
| PCT_SE_T159_002 | 0.153944 | 1.166426 | 0.220255 | 0.698935 | 0.484593 | 0.757482 | 1.796147 |  |
| vacanthouse | -0.28048 | 0.755422 | 0.22887 | -1.22549 | 0.22039 | 0.482361 | 1.183063 |  |
| PCT_SE_T027_002 | 0.024036 | 1.024327 | 0.226712 | 0.10602 | 0.915566 | 0.656836 | 1.597424 |  |
| PCT_SE_T027_003 | 0.010163 | 1.010215 | 0.217756 | 0.04667 | 0.962776 | 0.659259 | 1.548 |  |
| hscollegegrad | 0.089822 | 1.093979 | 0.202092 | 0.444458 | 0.656711 | 0.736181 | 1.625674 |  |
| morecollege | 0.026423 | 1.026775 | 0.202426 | 0.130532 | 0.896146 | 0.690505 | 1.526807 |  |
| PCT_SE_T069_005 | 0.221303 | 1.247701 | 0.203541 | 1.087266 | 0.276919 | 0.837246 | 1.859379 |  |
| SE_T070_002 | -0.05868 | 0.943013 | 0.272692 | -0.21517 | 0.829634 | 0.552583 | 1.609303 |  |
| PCT_SE_T070_006 | -0.14388 | 0.865989 | 0.235841 | -0.61009 | 0.541805 | 0.545456 | 1.374879 |  |
| workingclass | 0.000409 | 1.000409 | 0.209519 | 0.001951 | 0.998443 | 0.663486 | 1.508424 |  |
| PCT_SE_T086_002 | -0.0437 | 0.957241 | 0.23029 | -0.18976 | 0.849496 | 0.609529 | 1.503308 |  |
| PCT_SE_T086_003 | 0.020944 | 1.021165 | 0.199084 | 0.105201 | 0.916216 | 0.691245 | 1.50855 |  |
| bluecollar | -0.01531 | 0.984803 | 0.248416 | -0.06165 | 0.950846 | 0.605193 | 1.602526 |  |
| PCT_SE_T086_012 | 0.280131 | 1.323303 | 0.211189 | 1.326444 | 0.184693 | 0.874765 | 2.001829 |  |
| PCT_SF1_P020012 | -0.01172 | 0.988352 | 0.230809 | -0.05076 | 0.959514 | 0.6287 | 1.553745 |  |
| PCT_SF1_P010010 | -0.26338 | 0.768448 | 0.232733 | -1.13169 | 0.257764 | 0.486977 | 1.212609 |  |
| PCT_SF1_P010011 | 0.020584 | 1.020797 | 0.204174 | 0.100814 | 0.919698 | 0.684136 | 1.523127 |  |
| PCT_SF1_P012B002 | -0.84703 | 0.428687 | 0.418574 | -2.02361 | 0.043011 | 0.188731 | 0.973728 | * |
| PCT_SF1_H003003 | -0.22553 | 0.79809 | 0.220238 | -1.02404 | 0.305815 | 0.5183 | 1.228917 |  |
| PCT_SF3_P064002 | -0.20483 | 0.814789 | 0.203183 | -1.00808 | 0.313414 | 0.547132 | 1.213384 |  |
| hhincome15k | -0.0458 | 0.955235 | 0.196667 | -0.23287 | 0.815863 | 0.649686 | 1.404484 |  |
| hhincome150k | -0.24345 | 0.783918 | 0.241839 | -1.00667 | 0.314095 | 0.487993 | 1.259296 |  |
| PCT_SF3_P052002 | -0.11777 | 0.888897 | 0.206358 | -0.57072 | 0.568187 | 0.593193 | 1.332009 |  |
| PCT_SF3_P052003 | -0.19812 | 0.820268 | 0.19653 | -1.00811 | 0.3134 | 0.558041 | 1.205716 |  |
| SF3_P077001 | -0.01145 | 0.988617 | 0.207882 | -0.05507 | 0.956082 | 0.657772 | 1.485871 |  |
| PCT_SF3_P089002 | -0.12249 | 0.884716 | 0.205023 | -0.59744 | 0.550214 | 0.59195 | 1.322278 |  |
| PCT_SF3_P089021 | 0.172628 | 1.188423 | 0.212289 | 0.813171 | 0.41612 | 0.783911 | 1.801671 |  |
| PCT_SF3_P090002 | -0.13177 | 0.876546 | 0.199904 | -0.65914 | 0.509803 | 0.592397 | 1.29699 |  |
| PCT_SF3_P092002 | -0.12737 | 0.880405 | 0.208147 | -0.61194 | 0.540578 | 0.58547 | 1.323916 |  |
| PCT_SF3_H020002 | 0.218005 | 1.243593 | 0.236219 | 0.922892 | 0.356063 | 0.782716 | 1.975842 |  |
| crowding | -0.40286 | 0.668405 | 0.231719 | -1.73858 | 0.082109 | 0.424421 | 1.052647 | * (LASSO) |
| homeprice | -0.072 | 0.930531 | 0.243806 | -0.29532 | 0.767751 | 0.57703 | 1.500592 |  |

1. **Model Comparison and Interpretation for LASSO**

|  | AIC | AUC 12 mo | AUC 24 mo | AUC 36 mo |
| --- | --- | --- | --- | --- |
| **Total Population:** |  |  |  |  |
| **Patient Model** | -58.2 | 0.86 | 0.8869 | 0.81 |
| **Patient plus Neighborhood Models** | -33.3 | 0.89 | 0.924 | 0.91 |
| **White Men** |  |  |  |  |
| **Patient Model** | -43.4 | 0.87 | 0.85 | 0.84 |
| **Patient plus Neighborhood Models** | -25.5 | 0.98 | 0.95 | 0.93 |
| **Black Men without a PCa family history** | - |  |  |  |
| **Patient Model** | -25.1 | 0.91 | 0.92 | 0.92 |
| **Patient plus Neighborhood Models** | -10.4 | 0.91 | 0.93 | 0.93 |
| **Black Men with a PCa family history** |  |  |  |  |
| **Patient Model** | -29.3 | 0.93 | 0.92 | 0.90 |
| **Patient plus Neighborhood Models** | -10.9 | 0.94 | 0.95 | 0.91 |
