# Supplementary material for: The effect of neighborhood social environment on prostate cancer development in black and white men at high risk for prostate cancer
Source: PLoS One. 2020 Aug 13;15(8):e0237332. doi: 10.1371/journal.pone.0237332 (PMC7425919; doi:10.1371/journal.pone.0237332)
Supplement: S3 Table — (DOCX) [file pone.0237332.s003.docx]

S3 Table. Correlation Analysis of Neighborhood Socioeconomic (nSES) Variables (Significant nSES Variables vs. All)

|  | PCT_SF3_H042006 | PCT_SF1_P012B002 | pct_sf3_p030007 | pct_sf3_p084006 | PCT_SE_T069_006_y |
| --- | --- | --- | --- | --- | --- |
| pct_sf3_p030007 | 0.11 | -0.34 | 1.00 | 0.22 | 0.42 |
| pct_sf3_p050026 | 0.47 | -0.04 | 0.22 | -0.05 | 0.08 |
| pct_sf3_p052012 | 0.36 | 0.29 | -0.24 | -0.30 | -0.46 |
| pct_sf3_p084006 | 0.01 | -0.08 | 0.22 | 1.00 | 0.33 |
| pct_sf3_p092021 | -0.12 | -0.25 | 0.28 | 0.37 | 0.56 |
| pct_sf3_p120002 | -0.05 | -0.39 | 0.44 | 0.43 | 0.76 |
| pct_sf3_p159i007 | -0.05 | -0.31 | 0.41 | 0.34 | 0.57 |
| pct_sf3_h019093 | -0.41 | -0.21 | 0.04 | 0.15 | 0.28 |
| pct_sf3_h045025 | -0.22 | -0.30 | 0.31 | 0.30 | 0.59 |
| pct_sf3_pct050102 | -0.10 | -0.29 | 0.37 | 0.37 | 0.66 |
| pct_sf3_pct051020 | -0.01 | 0.05 | -0.10 | 0.00 | -0.21 |
| pct_sf3_pct075a006 | 0.00 | 0.33 | -0.24 | 0.07 | -0.12 |
| pct_sf3_hct004093 | 0.03 | -0.20 | 0.28 | 0.27 | 0.45 |
| pct_sf3_hct005083 | -0.26 | -0.20 | 0.24 | 0.18 | 0.47 |
| pct_sf3_hct015042 | -0.10 | -0.27 | 0.30 | 0.35 | 0.54 |
| pct_sf3_hct017019 | -0.08 | -0.15 | 0.23 | 0.31 | 0.42 |
| pct_sf1_p030012 | 0.01 | -0.16 | 0.18 | 0.28 | 0.48 |
| PCT_SF3_H021045 | -0.32 | -0.34 | 0.26 | 0.29 | 0.51 |
| PCT_SF3_H111002 | 0.07 | -0.26 | 0.33 | 0.29 | 0.52 |
| PCT_SF3_PCT025051 | -0.06 | -0.07 | -0.04 | -0.09 | -0.07 |
| PCT_SF3_P033003 | -0.14 | -0.44 | 0.29 | 0.35 | 0.67 |
| PCT_SF3_H042006 | 1.00 | 0.08 | 0.11 | 0.01 | -0.05 |
| PCT_SF3_P010015 | 0.07 | -0.38 | 0.42 | 0.33 | 0.67 |
| PCT_SE_T059_002 | 0.08 | -0.02 | 0.16 | 0.21 | 0.39 |
| popzeronine | 0.02 | 0.02 | 0.06 | 0.09 | 0.17 |
| poptennineteen | 0.01 | -0.01 | 0.19 | 0.22 | 0.32 |
| SF3_P053001 | 0.02 | 0.27 | -0.33 | -0.44 | -0.65 |
| SF3_H085001 | -0.19 | 0.23 | -0.39 | -0.42 | -0.66 |
| novehicle | -0.06 | -0.42 | 0.54 | 0.41 | 0.73 |
| PCT_SF3_P087002 | -0.09 | -0.34 | 0.38 | 0.44 | 0.72 |
| PCT_SF1_P007003 | 0.01 | -0.52 | 0.51 | 0.29 | 0.71 |
| PCT_SF1_P007002 | 0.00 | 0.51 | -0.51 | -0.30 | -0.71 |
| PCT_SF1_P010009 | -0.14 | -0.01 | -0.04 | 0.30 | 0.25 |
| PCT_SF1_P007007 | -0.14 | 0.01 | -0.01 | 0.30 | 0.26 |
| PCT_SE_T069_006_y | -0.05 | -0.37 | 0.42 | 0.33 | 1.00 |
| collegeover25_y | -0.28 | 0.08 | -0.32 | -0.43 | -0.58 |
| PCT_SE_T040_002_y | 0.12 | -0.17 | 0.31 | 0.45 | 0.62 |
| PCT_SE_T085_017 | -0.27 | 0.08 | -0.26 | -0.33 | -0.53 |
| PCT_SF3_P018013 | 0.04 | 0.39 | -0.41 | -0.37 | -0.66 |
| PCT_SF3_P021013 | -0.07 | -0.02 | -0.05 | 0.11 | -0.05 |
| SF1_P037002 | -0.20 | -0.03 | -0.09 | -0.06 | -0.13 |
| houses1970 | -0.23 | 0.40 | -0.41 | -0.31 | -0.63 |
| PCT_SF3_H034009 | 0.28 | -0.37 | 0.39 | 0.33 | 0.59 |
| PCT_SF3_H038002 | 0.51 | 0.32 | -0.20 | -0.30 | -0.51 |
| PCT_SF3_H047003 | -0.03 | -0.11 | 0.30 | 0.26 | 0.44 |
| PCT_SF3_P115007 | 0.55 | -0.06 | 0.17 | 0.02 | -0.02 |
| PCT_SE_T159_002 | 0.53 | 0.20 | -0.08 | -0.15 | -0.28 |
| vacanthouse | -0.21 | -0.18 | 0.39 | 0.37 | 0.61 |
| PCT_SE_T027_002 | -0.07 | -0.43 | 0.42 | 0.38 | 0.69 |
| PCT_SE_T027_003 | 0.06 | 0.44 | -0.44 | -0.37 | -0.71 |
| hscollegegrad | -0.14 | 0.23 | -0.41 | -0.44 | -0.67 |
| morecollege | -0.32 | 0.04 | -0.29 | -0.37 | -0.51 |
| PCT_SE_T069_005 | 0.10 | 0.37 | -0.33 | -0.41 | -0.64 |
| SE_T070_002 | 0.21 | 0.28 | -0.20 | -0.21 | -0.39 |
| PCT_SE_T070_006 | -0.10 | -0.40 | 0.41 | 0.34 | 0.92 |
| workingclass | 0.24 | -0.05 | 0.26 | 0.46 | 0.54 |
| PCT_SE_T086_002 | -0.18 | 0.17 | -0.32 | -0.44 | -0.59 |
| PCT_SE_T086_003 | -0.21 | -0.04 | -0.19 | -0.41 | -0.46 |
| bluecollar | 0.31 | 0.23 | 0.01 | 0.34 | 0.20 |
| PCT_SE_T086_012 | 0.40 | 0.33 | 0.00 | 0.11 | -0.11 |
| PCT_SF1_P020012 | 0.07 | -0.37 | 0.42 | 0.32 | 0.68 |
| PCT_SF1_P010010 | -0.17 | 0.17 | -0.31 | 0.15 | -0.05 |
| PCT_SF1_P010011 | -0.01 | -0.35 | 0.34 | 0.31 | 0.65 |
| PCT_SF1_P012B002 | 0.08 | 1.00 | -0.34 | -0.08 | -0.37 |
| PCT_SF1_H003003 | -0.21 | -0.19 | 0.38 | 0.37 | 0.62 |
| PCT_SF3_P064002 | -0.02 | -0.31 | 0.44 | 0.40 | 0.73 |
| hhincome15k | -0.08 | -0.30 | 0.33 | 0.44 | 0.66 |
| hhincome150k | -0.24 | 0.17 | -0.33 | -0.35 | -0.58 |
| PCT_SF3_P052002 | -0.08 | -0.37 | 0.36 | 0.41 | 0.69 |
| PCT_SF3_P052003 | -0.06 | -0.14 | 0.19 | 0.42 | 0.51 |
| SF3_P077001 | -0.07 | 0.24 | -0.35 | -0.44 | -0.65 |
| PCT_SF3_P089002 | -0.09 | -0.34 | 0.38 | 0.44 | 0.72 |
| PCT_SF3_P089021 | 0.09 | 0.34 | -0.38 | -0.44 | -0.72 |
| PCT_SF3_P090002 | -0.01 | -0.30 | 0.34 | 0.43 | 0.68 |
| PCT_SF3_P092002 | -0.09 | -0.36 | 0.38 | 0.43 | 0.71 |
| PCT_SF3_H020002 | 0.51 | 0.32 | -0.20 | -0.30 | -0.51 |
| crowding | -0.20 | -0.31 | 0.27 | 0.41 | 0.60 |
| homeprice | -0.27 | 0.14 | -0.30 | -0.28 | -0.48 |
