# Supplementary material for: The effect of neighborhood social environment on prostate cancer development in black and white men at high risk for prostate cancer
Source: PLoS One. 2020 Aug 13;15(8):e0237332. doi: 10.1371/journal.pone.0237332 (PMC7425919; doi:10.1371/journal.pone.0237332)

S3 Figure. Correlation Analysis of Neighborhood Socioeconomic (nSES) Variables (Significant nSES Variables vs. All)


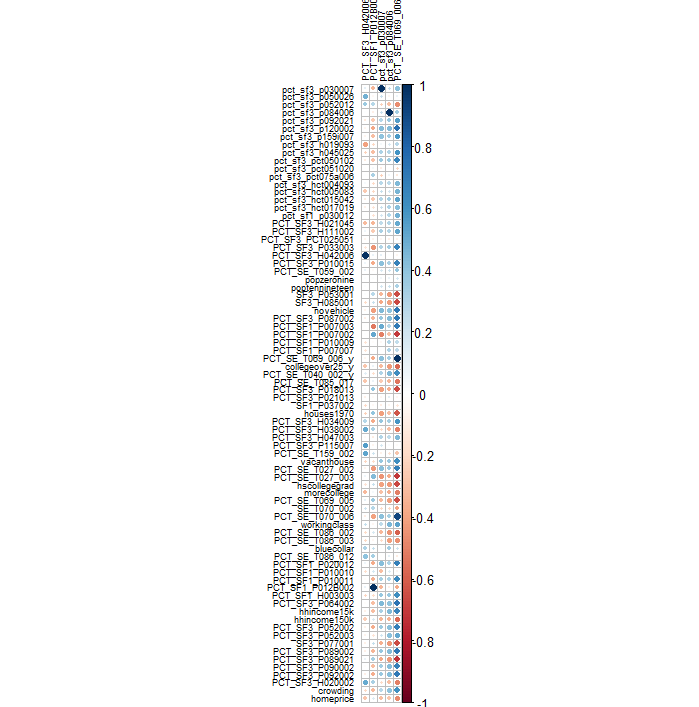

Supplement: S1 Fig — (DOCX) [file pone.0237332.s004.docx]
